# Effect of Physical Activity Coaching on Acute Care and Survival Among Patients With Chronic Obstructive Pulmonary Disease: A Pragmatic Randomized Clinical Trial

**DOI:** 10.1001/jamanetworkopen.2019.9657

**Published:** 2019-08-16

**Authors:** Huong Q. Nguyen, Marilyn L. Moy, In-Lu Amy Liu, Vincent S. Fan, Michael K. Gould, Smita A. Desai, William J. Towner, George Yuen, Janet S. Lee, Stacy J. Park, Anny H. Xiang

**Affiliations:** 1 Department of Research and Evaluation, Kaiser Permanente Southern California, Pasadena; 2 Harvard Medical School, Boston, Massachusetts; 3 VA Boston Healthcare System, Boston, Massachusetts; 4 University of Washington, Seattle; 5 VA Puget Sound Health Care System, Seattle, Washington; 6 Kaiser Permanente Southern California, San Diego; 7 Kaiser Permanente Southern California, Orange County, Anaheim

## Abstract

**Importance:**

While observational studies show that physical inactivity is associated with worse outcomes in chronic obstructive pulmonary disease (COPD), there are no population-based trials to date testing the effectiveness of physical activity (PA) interventions to reduce acute care use or improve survival.

**Objective:**

To evaluate the long-term effectiveness of a community-based PA coaching intervention in patients with COPD.

**Design, Setting, and Participants:**

Pragmatic randomized clinical trial with preconsent randomization to the 12-month Walk On! (WO) intervention or standard care (SC). Enrollment occurred from July 1, 2015, to July 31, 2017; follow-up ended in July 2018. The setting was Kaiser Permanente Southern California sites. Participants were patients 40 years or older who had any COPD-related acute care use in the previous 12 months; only patients assigned to WO were approached for consent to participate in intervention activities.

**Interventions:**

The WO intervention included collaborative monitoring of PA step counts, semiautomated step goal recommendations, individualized reinforcement, and peer/family support. Standard COPD care could include referrals to pulmonary rehabilitation.

**Main Outcomes and Measures:**

The primary outcome was a composite binary measure of all-cause hospitalizations, observation stays, emergency department visits, and death using adjusted logistic regression in the 12 months after randomization. Secondary outcomes included self-reported PA, COPD-related acute care use, symptoms, quality of life, and cardiometabolic markers.

**Results:**

All 2707 eligible patients (baseline mean [SD] age, 72 [10] years; 53.7% female; 74.3% of white race/ethnicity; and baseline mean [SD] percent forced expiratory volume in the first second of expiration predicted, 61.0 [22.5]) were randomly assigned to WO (n = 1358) or SC (n = 1349). The intent-to-treat analysis showed no differences between WO and SC on the primary all-cause composite outcome (odds ratio [OR], 1.09; 95% CI, 0.92-1.28; *P* = .33) or in the individual outcomes. Prespecified, as-treated analyses compared outcomes between all SC and 321 WO patients who participated in any intervention activities (23.6% [321 of 1358] uptake). The as-treated, propensity score–weighted model showed nonsignificant positive estimates in favor of WO participants compared with SC on all-cause hospitalizations (OR, 0.84; 95% CI, 0.65-1.10; *P* = .21) and death (OR, 0.62; 95% CI, 0.35-1.11; *P* = .11). More WO participants reported engaging in PA compared with SC (47.4% [152 of 321] vs 30.7% [414 of 1349]; *P* < .001) and had improvements in the Patient-Reported Outcomes Measurement Information System 10 physical health domain at 6 months. There were no group differences in other secondary outcomes.

**Conclusions and Relevance:**

Participation in a PA coaching program by patients with a history of COPD exacerbations was insufficient to effect improvements in acute care use or survival in the primary analysis.

**Trial Registration:**

ClinicalTrials.gov identifier: NCT02478359

## Key Points

**Question:**

What is the real-world effectiveness of a 12-month community-based physical activity (PA) coaching intervention on reducing all-cause acute care use and death in patients with a history of a chronic obstructive pulmonary disease (COPD) exacerbation?

**Findings:**

In this multisite, randomized clinical trial that included a population-based sample of 2707 patients with COPD, 321 of 1358 patients participated in the PA coaching intervention and increased PA significantly, but there were no significant differences in the all-cause primary outcome (compostite measure of all-cause hospitalizations, observation stays, emergency department visits, and death) at 12 months.

**Meaning:**

Most patients with a COPD exacerbation did not engage in PA, and the limited PA did not lead to significant benefit in 12-month health care use.

## Teaser

This pragmatic randomized clinical trial evaluates the long-term effectiveness of a community-based physical activity coaching intervention on acute care and survival in patients with chronic obstructive pulmonary disease.

## Introduction

Chronic obstructive pulmonary disease (COPD) is the fourth leading cause of death in the United States.^1^ Observational studies^2,3,4,5,6,7,8^ consistently show that physical inactivity is associated with increased hospitalizations and death. Moreover, it has previously been shown that hospitalized patients with COPD who engaged in physical activity (PA) before the index admission had a 34% lower risk of 30-day readmission compared with inactive patients.^9^

Despite the unequivocal evidence that pulmonary rehabilitation (PR) improves patient-centered outcomes,^10,11^ participation remains dismal at approximately 5%^12,13^ due to persistent barriers.^14,15^ Health systems need alternative approaches to achieve more patient-centered care, with better outcomes at lower cost, for the majority of patients who are not able to participate in PR, especially in light of current national PA guidelines^16^ and continued pressure to reduce readmissions.^17^ Small studies^18,19,20,21,22,23,24^ of PA interventions showed positive short-term to medium-term improvements in PA. The real-world and long-term effectiveness of interventions to increase PA and positively alter health care use in a large representative sample of older adults at high risk for recurrent COPD exacerbations remains unknown.

To address this gap, we conducted a pragmatic randomized clinical trial to determine the effectiveness of a 12-month community-based PA coaching program (Walk On! [WO]) that was designed to be generalizable, scalable, and sufficiently flexible to meet the needs of a large diverse sample of patients with a history of COPD exacerbations. The WO intervention was designed based on learnings from a series of collective studies^24,25,26,27^ by members of the investigative team that were informed by early and deep engagement with patient stakeholders and was grounded in social cognitive^28^ and self-regulation^29,30^ theories and core principles of motivational interviewing.^31^ We hypothesized that the WO intervention would increase PA and consequently reduce future acute care use and death in patients who have had a previous COPD exacerbation.

## Methods

### Study Design and Setting

This pragmatic randomized clinical trial compared the effectiveness of a 12-month PA intervention with standard care (SC) in patients with COPD. Written informed consent was obtained only from patients who were randomized to the WO intervention. The research question, design, and methods are aligned with methodological standards for pragmatic trials.^32,33^ This study was approved by the Kaiser Permanente Southern California Institutional Review Board. The study took place at 8 medical centers within a large integrated health system. This study followed the Consolidated Standards of Reporting Trials (CONSORT) reporting guideline.

### Study Population

English-speaking and Spanish-speaking patients with a COPD-related hospitalization, emergency department visit, or observation stay in the previous 12 months were identified from electronic medical records (EMRs). Patients were automatically excluded if the administrative and clinical EMR data showed that they had the following characteristics: they were younger than 40 years; they had a forced expiratory volume/forced vital capacity (FEV_1_/FVC) ratio exceeding 0.70; they were discharged to hospice or to another acute, postacute, or long-term care facility; they were nonambulatory at admission or discharge; they had Alzheimer disease, dementia, or metastatic cancer; they were morbidly obese (body mass index [calculated as weight in kilograms divided by height in meters squared] >40); or they had completed PR in the last 6 months. No medical record reviews or further screenings were conducted. Eligible patients were identified and randomized for the study from July 1, 2015, to July 31, 2017, and were followed up through July 2018. The setting was Kaiser Permanente Southern California sites.

### Randomization

A total of 2707 eligible patients were randomly assigned 1:1 to the WO intervention or SC, stratified by medical center, time from COPD acute care event (<6 vs ≥6 months), self-reported PA obtained from the exercise vital sign^34^ (inactive vs active), and median age (<72 vs ≥72 years) by random permuted block randomization. Patients randomized to WO received a recruitment letter and study brochure via postal mail and were followed up by telephone at least 10 business days after the mailing by existing clinical staff (respiratory therapists who served as the PA coaches) to assess their interest in WO. Following a single-consent, encouragement design,^35,36^ only those assigned to WO were approached for written consent to participate in intervention activities. Therefore, 2 groups were formed within WO, namely, WO participants and WO nonparticipants. Patients assigned to SC were not contacted about the study except for a random subgroup who were invited to complete surveys during 12 months. Detailed study methods and implementation learnings have been published.^37,38^ The trial protocol, including the scientific rationale for the study design, is available in Supplement 1.

### Study Interventions

The WO intervention included collaborative monitoring of PA step counts, semiautomated step goal recommendations, individualized reinforcement, and peer/family support, with built-in flexibility to accommodate the diverse preferences and needs of patients, as well as anticipated implementation constraints. Walking was promoted as a primary mode of PA because almost 90% of activities that patients with COPD engage in are ambulatory in nature and walking is a safe and accessible form of PA.^39^

The WO participants were sent a baseline previsit packet that included a consent form, activity sensor to wear for 7 days before the visit, and a survey packet (measures are described in the Study Outcomes subsection). During the baseline visit, the coach reviewed the survey responses, baseline PA, and performance on a 6-minute walk test to collaboratively design an individualized PA program for the patient, starting with an initial step goal for the first week.

Patients received 4 weekly coaching phone calls during weeks 1 through 5 to reinforce or readjust the PA plan as needed. Outreach by the PA coaches for the remaining 11 months were individualized and targeted according to patients’ progress with their walking program, technical difficulties, or automated triggers from the study dashboard based on data submitted by patients. Depending on whether patients chose to use a low-tech or internet-enabled activity device, they uploaded or reported their PA and symptoms on a weekly basis via an interactive phone voice response system or web interface. Step goal recommendations were provided by the respective systems. Patients were also encouraged to attend monthly group support meetings with their peer/family caregivers.

### Comparison Group

The SC patients continued to receive their routine care and had access to all health services in accordance with their health plan. Patients received no instructions to exercise and were not contacted about the trial except for a randomly selected subgroup (n = 537) to complete surveys for comparison with WO participants.

### Study Outcomes

The primary outcome was a composite binary measure of any all-cause hospitalizations, observation stays, emergency department visits, and death. Secondary outcomes included COPD-related acute care use, cardiometabolic markers (blood pressure, glycated hemoglobin, and lipids), and self-reported PA.^34^ Follow-up time was 12 months from the date of randomization. These outcomes were available for all patients from the EMRs. Additional secondary outcomes included the following: symptoms (COPD Assessment Test),^40^ depression (Patient Health Questionnaire 8),^41^ anxiety (Generalized Anxiety Disorder 7),^42^ health-related quality of life (Patient-Reported Outcomes Measurement Information System 10 [PROMIS-10]),^43^ and satisfaction with the program for a subset of the sample. These data were obtained from mailed surveys for all WO participants and a randomly selected subset of SC patients at baseline, 6 months, and 12 months.

### Sample Size and Statistical Analysis

Sample size was calculated based on estimates of acute care use and death from previous studies^9,44,45,46^ and with the assumption that approximately 50% of the patients assigned to WO would participate in the intervention. Allowing for a 15% disenrollment from the health plan and 2-tailed α = .05, we anticipated that by enrolling a total of 1650 patients we would have 80% power to detect an absolute reduction of 7% in the primary composite outcome (70% vs 63%). We reached the enrollment target after 12 months, but the participation rate remained low at 23.5% (147 of 625). The data and safety monitoring board approved a revised power calculation based on a projected accrual of 2700 patients through the end of the original 24-month recruitment timeline to detect a smaller difference of 5.5% (see eMethods in Supplement 2).

The primary analyses followed the intent-to-treat (ITT) principle such that all randomized patients regardless of participation were included. Follow-up time was 12 months from the date of randomization. Secondary analyses included prespecified, as-treated analyses in which patients who participated in WO were compared with SC, and follow-up time was 2 to 12 months from the date of randomization. The first 2 months after randomization were excluded because it was expected that it would take approximately 2 months from the date of randomization to start the intervention. Therefore, only patients who were followed up for at least 2 months across both arms from the date of randomization were eligible for the as-treated analysis.

For the intervention effects on outcomes, prespecified analyses used adjusted logistic regression as the primary approach for any occurrence of the outcomes within 12 months and used survival analysis as the secondary approach for time to the first occurrence of each outcome. Disenrollment rates were slightly higher in SC vs WO (7.9% [107 of 1349] vs 4.8% [65 of 1359]). For patients who disenrolled from the health plan, data before disenrollment were used, and the shorter duration of follow-up was incorporated into the survival analysis for all patients. All patients had data on primary outcomes after randomization. For the as-treated analysis, stabilized (standardized difference, <0.1) propensity score inverse probability of treatment weighting^47^ was used to balance baseline characteristics between patients who participated in WO and the SC group. The propensity of being a WO participant was calculated using a logistic regression model of baseline sociodemographics, health behaviors, disease severity, comorbidities, inhalers/medications, health care use in the prior year, and study site.

The a priori threshold for statistical significance was a 2-sided *P* < .05. Results for the secondary analyses should be interpreted as exploratory due to multiple comparisons. All analyses were conducted using SAS, version 9.4 for Windows statistical software (SAS Institute Inc).

## Results

### Patient Flow and Baseline Characteristics

A total of 2707 patients (baseline mean [SD] age, 72 [10] years; 53.7% female; 74.3% of white race/ethnicity; and baseline mean [SD] forced expiratory volume in the first second of expiration (FEV_1_%) predicted, 61.0 [22.5]) were identified from the EMRs and randomized to WO (n = 1358) or SC (n = 1349). Only 321 of 1358 patients (23.6%) randomized to WO consented to participate in the intervention components and completed baseline surveys (Figure). In total, 471 of 1358 patients (34.6%) expressed no interest in the WO program. An additional 313 of 1358 patients (23.0%) passively declined to participate (patients who did not return our telephone calls despite 3 contacts). A total of 268 of 537 randomly sampled SC patients (49.9%) completed baseline surveys. Baseline characteristics were similar between all randomized WO and SC patients (Table 1 and eTable 1 in Supplement 2). In those randomized to WO, there were significant baseline differences between WO participants vs nonparticipants (fewer current smokers, greater use of long-acting inhalers, fewer comorbidities, fewer all-cause emergency department visits, more outpatient-treated COPD exacerbations, and greater use of specialty care).

**Figure.**
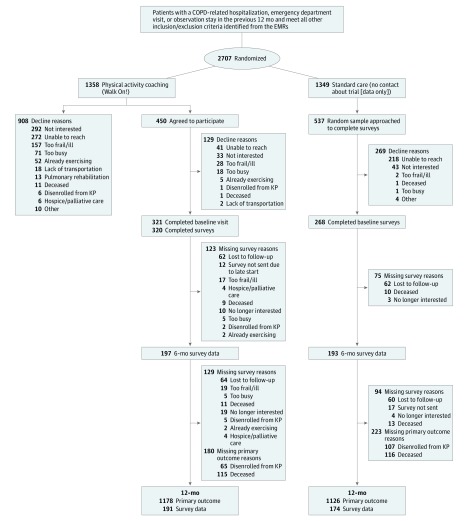
CONSORT Patient Flow CONSORT indicates Consolidated Standards of Reporting Trials; COPD, chronic obstructive pulmonary disease; EMRs, electronic medical records; and KP, Kaiser Permanente.

**Table 1.** Baseline Sample Characteristics^a^

Characteristic	Participants, No. (%)			*P* Value for SC vs WO	Participants, No. (%)		*P* Value for WO-P vs WO-Non-P
	Total (N = 2707)	Standard Care (n = 1349)	WO (n = 1358)		WO-P (n = 321)	WO-Non-P (n = 1037)	
Age, mean (SD), y	72 (10)	72 (10)	72 (10)	.98	72 (9)	73 (11)	.12
Female	1455 (53.7)	739 (54.8)	716 (52.7)	.28	175 (54.5)	541 (52.2)	.46
Marital status							.36
Partnered	1343 (49.6)	677 (50.2)	666 (49.0)	.57	163 (50.8)	503 (48.5)	
Education							
College degree or higher	665 (24.6)	329 (24.4)	336 (24.7)	.95	79 (24.6)	257 (24.8)	.99
Some college	869 (32.1)	432 (32.0)	437 (32.2)		105 (32.7)	332 (32.0)	
High school or less	1154 (42.6)	579 (42.9)	575 (42.3)		137 (42.7)	438 (42.2)	
Median household income							
<$50 000	1236 (45.7)	616 (45.7)	620 (45.7)	.99	145 (45.2)	474 (45.7)	.76
≥$50 000	1452 (53.6)	724 (53.7)	728 (53.6)		176 (54.8)	553 (53.3)	
Race/ethnicity							
American Indian/Alaska Native	8 (0.0)	4 (0.0)	4 (0.0)	.24	1 (0.0)	3 (0.0)	.31
Asian	140 (5.2)	59 (4.4)	81 (6.0)		16 (5.0)	65 (6.3)	
Black/African American	375 (13.9)	188 (13.9)	187 (13.8)		41 (12.8)	146 (14.1)	
Hawaiian/Pacific Islander	19 (0.7)	6 (0.0)	13 (0.9)		2 (0.6)	11 (1.0)	
White	2010 (74.3)	1021 (75.7)	989 (72.8)		245 (76.3)	744 (71.7)	
Multirace/multiethnicity	7 (0.0)	4 (0.0)	3 (0.0)		2 (0.6)	1 (0.0)	
Other/unknown	148 (5.4)	67 (5.0)	81 (6.0)		14 (4.4)	67 (6.5)	
Smoking status							
Never	331 (12.2)	162 (12.0)	169 (12.4)	.85	43 (13.4)	126 (12.2)	.002
Former	1868 (69.0)	933 (69.2)	935 (68.9)		242 (75.4)	693 (66.8)	
Current	481 (17.8)	243 (18.0)	238 (17.5)		34 (10.6)	204 (19.7)	
Spirometry, most recent							
No.	2036	996	1040		269	771	
FEV_1_/FVC, mean (SD)	55.1 (14.3)	54.8 (14.7)	55.4 (13.9)	.36	53.5 (13.9)	56.0 (13.9)	.009
FEV_1_% predicted, mean (SD)	61.0 (22.5)	60.0 (21.9)	62.1 (23.1)	.05	59.3 (22.1)	63.1 (23.3)	.02
GOLD I, ≥80%	406 (19.9)	185 (18.6)	221 (21.3)	.29	49 (18.2)	172 (22.3)	.14
GOLD II, 50% to <80%	857 (42.1)	417 (41.9)	440 (42.3)		109 (40.5)	331 (42.9)	
GOLD III, 30% to <50%	563 (27.7)	288 (28.9)	275 (26.4)		85 (31.6)	190 (24.6)	
GOLD IV, <30%	131 (6.4)	69 (6.9)	62 (6.0)		16 (5.9)	46 (6.0)	
Medications							
Long-acting β-2 agonist	1646 (60.8)	846 (62.7)	800 (58.9)	.04	212 (66.0)	588 (56.7)	.01
Long-acting anticholinergic	1422 (52.5)	702 (52.0)	720 (53.0)	.58	198 (61.7)	522 (50.3)	.002
LAMA and ICS	1244 (46.0)	631 (46.8)	613 (45.1)	.40	170 (53.0)	443 (42.7)	.004
LABA and ICS	1641 (60.6)	844 (62.6)	797 (58.7)	.04	210 (65.4)	587 (56.6)	.02
Long-term systemic corticosteroids	243 (9.0)	131 (9.7)	112 (8.2)	.19	31 (9.7)	81 (7.8)	.36
Oxygen use	1059 (39.1)	550 (40.8)	509 (37.5)	.08	125 (38.9)	384 (37.0)	.54
Charlson Comorbidity Index, mean (SD)	3.7 (2.3)	3.7 (2.2)	3.7 (2.3)	.38	3.4 (2.2)	3.8 (2.3)	.009
Heart failure	805 (29.7)	398 (29.5)	407 (30.0)	.79	78 (24.3)	329 (31.7)	.01
Pulmonary hypertension	143 (5.3)	70 (5.2)	73 (5.4)	.83	14 (4.4)	59 (5.7)	.36
Type 1 or type 2 diabetes	873 (32.2)	456 (33.8)	417 (30.7)	.08	100 (31.2)	317 (30.6)	.84
Depression	741 (27.4)	361 (26.8)	380 (28.0)	.48	86 (26.8)	294 (28.4)	.59
Anxiety	742 (27.4)	383 (28.4)	359 (26.4)	.25	80 (24.9)	279 (26.9)	.48
Chronic pain	545 (20.1)	273 (20.2)	272 (20.0)	.89	63 (19.6)	209 (20.2)	.84
**Health Care Use in Prior Year**							
All cause							
Hospitalizations	1437 (53.1)	735 (54.5)	702 (51.7)	.15	166 (51.7)	536 (51.7)	.99
Observation stays	786 (29.0)	394 (29.2)	392 (28.9)	.85	84 (26.2)	308 (29.7)	.22
Emergency department visits	2089 (77.2)	1050 (77.8)	1039 (76.5)	.41	229 (71.3)	810 (78.1)	.01
COPD-related							
Hospitalizations	1107 (40.9)	568 (42.1)	539 (39.7)	.16	134 (41.7)	405 (39.1)	.43
Observation stays	480 (17.7)	243 (18.0)	237 (17.5)	.66	50 (15.6)	187 (18.0)	.29
Emergency department visits	1531 (56.6)	766 (56.8)	765 (56.3)	.70	173 (53.9)	592 (57.1)	.25
Outpatient-treated COPD exacerbations	1902 (70.3)	966 (71.6)	936 (68.9)	.13	246 (76.6)	690 (66.5)	<.001

Abbreviations: COPD, chronic obstructive pulmonary disease; FEV_1_%, forced expiratory volume in the first second of expiration; FEV_1_/FVC, ratio of forced expiratory volume in the first second of expiration over forced vital capacity; GOLD, Global Initiative for Obstructive Lung Disease; ICS, inhaled corticosteroids; LABA, long-acting β-2 agonist; LAMA, long-acting anticholinergic; SC, standard care; WO, Walk On!; WO-P, Walk On! participants; WO-Non-P, Walk On! nonparticipants.

^a^ Baseline values were obtained in the 12 months before the cohort selection/randomization date; education and median household income were based on census data.

### WO Intervention Process Measures, PA, and Satisfaction

Approximately 83.3% (264 of 317) of WO participants completed at least 4 reinforcement telephone calls with the coaches in the first 5 weeks (eTable 2 in Supplement 2). The coaches’ documentation showed that they contacted and/or reviewed participants’ data on the study dashboard a median of 12 times during weeks 6 through 52. Almost one-quarter (72 of 317 [22.7%]) of the participants experienced technical challenges with their activity sensors, requiring at least 1 replacement, and 15.5% (50 of 321) chose not to use any of the study-issued devices. Less than half of the participants (n = 104 of 245) attended at least 1 optional monthly group visit. Participants who completed the 6-month and 12-month surveys provided favorable ratings of the program, with the vast majority reporting that it was easy to fit into their lifestyle (93.2% [165 of 177] on the 6-month survey and 97.1% [135 of 139] on the 12-month survey) and that they would recommend the program to others (97.8% [175 of 179] on the 6-month survey and 99.3 % [138 of 139] on the 12-month survey).

Step counts from any of the 3 activity sensors during 12 months showed that participants with higher step counts at baseline (≥5000 steps per day) tended to decline, whereas those with lower step counts at baseline (<5000 steps per day) remained steady (eFigure 1 in Supplement 2). Measures of self-reported PA (the exercise vital sign from the EMRs) in the 12 months after randomization were not significantly different between WO and SC groups in the ITT analysis (Table 2). However, there was a higher percentage of WO participants engaging in any PA (47.4% [152 of 321] vs 30.7% [414 of 1349]; *P* < .001) or meeting recommended PA levels (21.5% [69 of 321] vs 12.7% [171 of 1349]; *P* < .001) compared with SC.

**Table 2.** Changes in Physical Activity During 12 Months After Randomization

Exercise Vital Sign^a^	Participants, No. (%)						Adjusted *P* Values	
	Standard Care (n = 1349)		WO (n = 1358)		WO-P (n = 321)		SC vs WO^b^	SC vs WO-P^c^
	Baseline	12 mo	Baseline	12 mo	Baseline	12 mo		
Inactive, 0 min/wk	890 (66.0)	819 (60.7)	911 (67.1)	808 (59.5)	208 (64.8)	162 (50.5)	.34	<.001
Insufficiently active, 1-149 min/wk	262 (19.4)	243 (18.0)	245 (18.0)	268 (19.7)	67 (20.9)	83 (25.9)		
Active, ≥150 min/wk	160 (11.9)	171 (12.7)	174 (12.8)	182 (13.4)	43 (13.4)	69 (21.5)		
Missing^d^	37 (2.7)	116 (8.6)	28 (2.1)	100 (7.4)	3 (0.9)	7 (2.2)		
Any physical activity								
0 min/wk	927 (68.7)	935 (69.3)	939 (69.1)	908 (66.9)	211 (65.7)	169 (52.6)	.16	<.001
>0 min/wk	422 (31.3)	414 (30.7)	419 (30.9)	450 (33.1)	110 (34.3)	152 (47.4)		
Meet physical activity guidelines								
0-149 min/wk	1189 (88.1)	1178 (87.3)	1184 (87.2)	1176 (86.6)	278 (86.6)	252 (78.5)	.73	<.01
≥150 min/wk	160 (11.9)	171 (12.7)	174 (12.8)	182 (13.4)	43 (13.4)	69 (21.5)		

Abbreviations: SC, standard care; WO, Walk On!; WO-P, Walk On! participants.

^a^ Exercise vital sign values include all available data in the 12 months before and 12 months after the randomization date and are summarized as the median or modal value.

^b^ Intent-to-treat multinomial logistic regression analyses adjusted for age, forced expiratory volume in the first second of expiration predicted, Charlson Comorbidity Index, oxygen use, hospitalization for chronic obstructive pulmonary disease in the previous 12 months, outpatient-treated chronic obstructive pulmonary disease exacerbation in the previous 12 months, length of time since acute care use to randomization, use of long-acting β-2 agonist or inhaled corticosteroids, physical activity level, and study site.

^c^ As-treated multinomial logistic regression analyses used stabilized propensity score inverse probability of treatment weighting to balance baseline characteristics (sociodemographics, health behaviors, disease severity, comorbidities, inhalers/medications, clinical biomarkers, and health care use in the prior year) between patients who participated in Walk On! and the SC group.

^d^ Missing 12-month, postrandomization exercise vital sign for SC and WO (n = 216) due to not having an encounter during the year (85 [39.4%]) or exercise vital sign not captured during any encounter (131 [60.6%]).

### Primary ITT Findings for Acute Care Use and Death

For all randomized patients, there was no significant difference in the primary composite outcome of all-cause hospitalizations, observation stays, emergency department visits, and death between WO and SC in the 12 months after randomization (odds ratio [OR], 1.09; 95% CI, 0.92-1.28; *P* = .33) (Table 3). There were also no between-group differences in the individual primary outcomes or COPD-related acute care use (OR, 1.10; 95% CI, 0.93-1.31; *P* = .26). Time-to-first-event models similarly showed no differences between groups (eTable 3 and eFigure 2 in Supplement 2). The rate of falls with injuries was similar between groups. Prespecified interaction tests to assess whether the intervention effects differed by baseline morbidities, level of social support, race/ethnicity, age, sex, and internet access did not identify significant subgroup effects.

**Table 3.** Logistic Regression Analyses of the Walk On! Intervention on the Primary Composite Outcome of All-Cause Hospitalizations, Observation Stays, Emergency Department Visits, and Death

Health Care Use or Death	Participants, No. (%)		OR (95% CI)	
	Standard Care	Walk On!	Unadjusted	Adjusted
**Primary Intent-to-Treat Analysis: Follow-up for 12 mo After Randomization**^a^				
No.	1349	1358	NA	NA
All-cause acute care use and death	864 (64.0)	883 (65.0)	1.04 (0.89-1.22)	1.09 (0.92-1.28)
Hospitalizations	499 (37.0)	502 (36.9)	1.00 (0.85-1.17)	1.05 (0.89-1.24)
Observation stays	269 (19.9)	295 (21.7)	1.11 (0.93-1.34)	1.13 (0.93-1.37)
Emergency department visits	694 (51.4)	702 (51.7)	1.01 (0.87-1.17)	1.03 (0.88-1.20)
Death	117 (8.7)	117 (8.6)	0.99 (0.76-1.30)	1.02 (0.77-1.36)
COPD-related acute care use^b^	398 (29.5)	411 (30.3)	1.04 (0.88-1.22)	1.10 (0.93-1.31)
**Prespecified, As-Treated, IPTW Analysis: Follow-up for 2-12 mo After Randomization**^c^				
No.	1310^d^	321	NA	NA
All-cause acute care use and death	781 (59.6)	185 (57.6)	0.92 (0.72-1.18)	1.05 (0.82-1.35)
Hospitalizations	433 (33.1)	91 (28.3)	0.80 (0.61-1.05)	0.84 (0.65-1.10)
Observation stays	230 (17.6)	53 (16.5)	0.93 (0.67-1.29)	0.92 (0.66-1.28)
Emergency department visits	610 (46.6)	144 (44.9)	0.93 (0.72-1.19)	1.07 (0.84-1.36)
Death	95 (7.3)	13 (4.0)	0.54 (0.30-0.98)	0.62 (0.35-1.11)
COPD-related acute care use^b^	195 (14.9)	48 (15.0)	1.01 (0.71-1.42)	0.96 (0.68-1.35)

Abbreviations: COPD, chronic obstructive pulmonary disease; IPTW, inverse probability of treatment weighting; NA, not applicable; OR, odds ratio.

^a^ Intent to treat: adjusted ORs are from logistic regression models that included age, forced expiratory volume in the first second of expiration predicted, Charlson Comorbidity Index, oxygen use, hospitalization for COPD in the previous 12 months, outpatient-treated COPD exacerbation in the previous 12 months, length of time since acute care use to randomization, use of long-acting β-2 agonist or inhaled corticosteroids, physical activity level, and study site.

^b^ The COPD-related acute care use includes hospitalizations, observation stays, and emergency department visits for COPD exacerbations.

^c^ As treated: adjusted ORs are from logistic regression models that included stabilized propensity score IPTW to balance baseline characteristics (sociodemographics, health behaviors, disease severity, comorbidities, inhalers/medications, and health care use in the prior year) between patients who participated in Walk On! and the standard care group.

^d^ Standard care patients not included in the as-treated analysis were due to disenrollment (n = 17) and deaths (n = 22) in the first 2 months after randomization.

### As-Treated Findings for Acute Care Use and Death

During 10 months of follow-up, there were non–statistically significant positive estimates in favor of WO participants compared with all SC patients on all-cause hospitalizations (OR, 0.84; 95% CI, 0.65-1.10; *P* = .21) and death (OR, 0.62; 95% CI, 0.35-1.11; *P* = .11) (Table 3). Adjusted time-to-first-event analyses showed similar estimates, with significant group differences in observation stays (hazard ratio, 0.72; 95% CI, 0.53-0.98; *P* = .04) (eTable 3 and eFigure 3 in Supplement 2).

### Secondary Outcomes

There were no differences between WO participants and SC survey responders on the baseline to 6-month or 12-month changes in the COPD Assessment Test, Patient Health Questionnaire 8, or Generalized Anxiety Disorder 7. Exceptions were the PROMIS-10 physical health domain (effect size, 0.25; 95% CI, 0.05-0.45; *P* = .01) and sedentary time (effect size, −0.26; 95% CI, −0.48 to −0.04; *P* = .02) at 6 months (Table 4). There were no significant group differences in cardiometabolic markers for both the as-treated analyses and the ITT analyses (eTable 4 in Supplement 2).

**Table 4.** Patient-Reported Outcomes Change Scores From Baseline to 6 Months and 12 Months

Outcome^a^	Walk On! Participants			Standard Care Survey Responders			Difference (95% CI) Between Change Scores	*P* Value	Effect Size (95% CI)
	No.	Baseline, Mean (SD)	Mean Change From Baseline	No.	Baseline, Mean (SD)	Mean Change From Baseline			
**CAT, ↓0-40**									
6 mo	197	18.4 (7.4)	−0.44	193	20.3 (8.3)	−0.38	−0.06 (−1.3 to 1.19)	.93	−0.01 (−0.21 to 0.19)
12 mo	188	18.0 (7.3)	−0.15	171	20.5 (8.5)	−0.56	0.41 (−0.92 to 1.72)	.55	0.06 (−0.14 to 0.27)
**PHQ-8, ↓0-24**									
6 mo	186	5.1 (4.4)	−0.56	188	6.5 (5.8)	−0.56	0.00 (−0.83 to 0.86)	.97	0.00 (−0.20 to 0.21)
12 mo	183	4.9 (4.2)	−0.06	164	6.6 (6.0)	−0.30	0.24 (−0.67 to 1.14)	.60	0.06 (−0.16 to 0.27)
**GAD-7, ↓0-21**									
6 mo	187	4.2 (4.2)	−0.23	185	5.4 (5.8)	−0.39	0.16 (−0.71 to 1.03)	.72	0.04 (−0.17 to 0.24)
12 mo	184	4.0 (4.1)	−0.21	164	5.2 (5.8)	−0.63	0.43 (−0.42 to 1.28)	.32	0.11 (−0.11 to 0.32)
**PROMIS-10 Mental Health, 21-68↑**									
6 mo	195	47.8 (8.5)	−0.26	196	45.6 (9.2)	0.40	−0.67 (−2.01 to 0.67)	.33	−0.10 (−0.30 to 0.10)
12 mo	189	47.7 (8.8)	0.26	172	45.3 (8.9)	0.69	−0.43 (−1.9 to 1.03)	.56	−0.06 (−0.27 to 0.15)
**PROMIS-10 Physical Health, 16-68↑**									
6 mo	195	41.0 (6.7)	1.01	196	40.0 (8.4)	−0.44	1.45 (0.29 to 2.60)	.01	0.25 (0.05 to 0.45)
12 mo	189	40.7 (7.0)	0.97	172	39.8 (8.6)	0.49	0.48 (−0.84 to 1.79)	.47	0.08 (−0.13 to 0.28)
**Sedentary Time, h/d**^b^									
6 mo	172	4.7 (3.1)	−0.67	158	5.1 (3.8)	0.08	−0.75 (−1.39 to −0.12)	.02	−0.26 (−0.48 to −0.04)
12 mo	169	4.8 (3.0)	−0.31	140	5.2 (3.9)	−0.07	−0.24 (−0.93 to 0.45)	.50	−0.08 (−0.30 to 0.15)

Abbreviations: CAT, COPD Assessment Test; GAD-7, Generalized Anxiety Disorder 7; PHQ-8, Patient Health Questionnaire 8; PROMIS-10, Patient-Reported Outcomes Measurement Information System 10.

^a^ Arrows indicate direction of better scores.

^b^ Sedentary time is based on the following: “In the last 7 days, please estimate the time you spent watching TV or videos on a typical day.”

## Discussion

In this multisite pragmatic randomized clinical trial, a 12-month community-based PA coaching intervention had no effect on the primary composite outcome of all-cause acute care use and death in patients at high risk for recurrent COPD exacerbations. The lack of effects is at least partly attributable to the suboptimal uptake, with only one-quarter (321 of 1358) of the randomized patients participating in the intervention, the low intervention intensity, and the reduced engagement during the 12 months. The prespecified, as-treated analyses, which included only patients who participated in any WO intervention component, showed nonsignificant estimates favoring WO for reduced acute care use and death compared with controls.

The null results from our ITT analyses are not very different from those of 2 recent, multicomponent COPD self-management studies.^48,49^ While both studies showed short-term reductions in COPD-related hospitalizations and/or emergency department visits at 6 months, neither showed a persistent effect at 12 months. Moreover, neither intervention had a significant influence on all-cause hospitalizations or death at 6 or 12 months. These 2 studies^48,49^ also differed from our study in several ways. Our study was focused on increasing PA and prioritizing generalizability by randomizing all patients who had any COPD-related acute care event in the previous 12 months, with few exclusions, relying solely on EMR data without performing medical record reviews. In contrast, these 2 efficacy studies^48,49^ conducted extensive medical record reviews and screenings before recruiting patients, while they were still hospitalized, and ultimately enrolled and randomized 22% to 30% of likely the most motivated patients. Acceptability of WO to patients and level of participation are essential components of the real-world effectiveness. If trial enrollment was limited to those who volunteered, findings regarding intervention acceptability or adherence at the population level would have limited scientific value and clinical operations utility for health systems in their decision to invest in such programs.

While WO participation fell short of our target, the 23.6% (321 of 1358) uptake is considerable when contrasted with participation in PR by Medicare beneficiaries (4%) with COPD^13^ or among those who had a recent COPD hospital discharge (2.7%)^12^ or among patients exposed to lighter-touch no-cost behavioral programs (2%).^50^ Nonetheless, health systems need to weigh the resources associated with broad outreach efforts and the yield in patient engagement, especially in response to the recent PA guidelines recommending that all older adults and those with chronic conditions engage in some level of PA.^16^ When contacted, approximately 34.6% (471 of 1358) of patients in our study expressed no interest in the WO program. However, the rate of disinterested patients would increase to 57.7% (784 of 1358) if passive declines (patients who did not return our calls despite 3 contacts) were included. With more persuasive advice from existing trusted health care professionals vs the constrained recruitment consenting language required of research studies, it is possible that participation in programs like WO might be higher. Alternatively, our experience may be as good as it gets, where up to one-half of the target population may not be activated and may choose not to engage in any PA outreach efforts due to other competing priorities. Better methods for identifying activated patients who are contemplating change are needed to yield a higher return.

The WO intervention had a favorable effect on the process outcome of PA (captured in routine care for all patients), as reflected by a greater proportion of WO participants reporting engagement in any PA or at least 150 minutes per week of PA (meeting national guidelines^16^) compared with controls. A recent European study^20^ testing a similar PA intervention also found significant improvements in PA in the as-treated analyses but not in the ITT analyses. In addition, there were modest but significant improvements in the PROMIS-10 physical health domain and sedentary time among WO participants at 6 months compared with a subset of control survey respondents, but this effect dissipated by 12 months due to decreased engagement over time, a common observation that is similar to previous PA studies.^18,51^ Together, these findings suggest that the PA coaching intervention positively affected self-reported PA, inactivity, and physical quality of life but may not have been of sufficient dose across the target population (and with limited power) to have a significant, consistent influence on more distal outcomes of acute care use and survival, as seen in the as-treated analyses.

An earlier observational study^52^ examining PA intensity and duration using accelerometer data found that low-intensity PA had a protective effect on risk of COPD hospitalization, with no benefit from high-intensity PA. In fact, for every 1000-step increase in low-intensity PA, there was a 20% risk reduction in COPD hospitalizations.^52^ In our study, although the risk estimate for the as-treated analysis of COPD-related acute care use was in a favorable direction, the significant finding with all-cause observation stays suggests that being physically active may be associated with fewer, less severe hospital encounters that do not require an admission. Because use of observation stays is a new phenomenon in response to the Hospital Readmissions Reduction Program, there is little published research on how interventions alter these encounters in patients with COPD.^53,54,55^

### Strengths and Limitations

There are several strengths to our study. To our knowledge, this is the first large-scale, health systems effort to engage a diverse and broadly representative sample of patients at high risk for recurrent COPD exacerbations to address a key question of uptake and long-term effectiveness of a scalable and affordable approach to PA promotion. The WO intervention was intentionally designed to be easily integrated in patients’ daily lives. This tailored approach of encouraging increased PA based on individual needs and preferences is also consistent with the primary PA guideline recommendations.^16^

Our study also had limitations. Given the need for automated identification and randomization of eligible patients using EMR data to ensure broadest representation, we could not rely on spirometry as an inclusion criterion and may have included some patients without COPD. More importantly, our goal was to align with the real-world policy and practice, relying solely on the administrative and clinical data from the EMRs to identify eligible patients.^56^ However, our decision to be broadly inclusive of patients who may have been experiencing rapid decline in their health and who may have not benefited from the intervention likely contributed to the low uptake and engagement over time.

Despite our attempts to accommodate patient preferences by offering and integrating the 3 activity sensors into our system, the multiple technical challenges with the devices likely contributed to disengagement by some participants even though the coaches encouraged patients to prioritize accumulating PA time instead of their step counts when they had technical difficulties. While the individualized WO intervention may be perceived as being too heterogeneous, not being intensive or multifaceted, or not having rigorous fidelity control, the goal of the study was to work within the real-world constraints to test a scalable and affordable approach to promoting PA using existing frontline clinical staff. We relied on the exercise vital sign, a self-reported measure obtained from routine care, as a key process outcome because it was not practical to measure objective PA across all patients. The high level of missingness in the patient-reported outcomes and the multiple comparisons suggest caution in interpreting the significant PROMIS-10 findings. Despite our efforts to adjust for selection bias in the as-treated analyses, residual confounding is an important limitation.

The study draws from a population of insured patients from an integrated system, potentially limiting generalizability. Compared with other recently published COPD studies,^48^ our study sample appeared to be already optimized with their COPD, cardiovascular, and diabetic care, as reflected in the prescribed inhalers and cardiometabolic markers, with potentially little room for additional change. This is well illustrated with the attenuated effects of cardiac rehabilitation on death and cardiac events in recent trials compared with earlier studies, likely due to therapeutic advances.^57^

## Conclusions

In this pragmatic randomized clinical trial of patients at high risk for recurrent COPD exacerbations, a potentially scalable and sustainable 12-month PA coaching intervention was associated with increased self-reported PA and modest improvements in the PROMIS-10 physical quality of life domain but had no significant effects on the primary composite outcome of acute care use and death for both as-treated analyses and ITT analyses. Findings from this study call for more realistic expectations for frail, older patients with serious chronic conditions to engage in and meet PA recommendations and for increased PA to have an influence on distal outcomes. Future methods work is needed to more efficiently identify activated patients for behavioral interventions.

## Notes

Supplement 1. **Trial Protocol**

Supplement 2. **eMethods.** Additional Statistical Analysis Information **eTable 1.** Additional Baseline Characteristics **eTable 2.** Walk On! Participants Process and Satisfaction Measures **eTable 3.** Time-to-Event Analyses of the Walk On! Intervention on the Primary Composite Outcome of All-Cause Hospitalizations, Observation Stays, Emergency Department Visits, and Death **eTable 4.** Cardio-metabolic Markers Post-randomization **eFigure 1.** Steps per Day Change Over 12 Months for Walk On! Participants Who Shared Their Data, by Level of Functioning at Baseline **eFigure 2.** Intent-to-Treat Adjusted Time-to-Event Analyses of the Walk On! Intervention on the Primary Composite Outcome of All-Cause Hospitalizations, Observation Stays, Emergency Department Visits, and Death 12-Months Post-randomization **eFigure 3.** As-Treated Adjusted Time-to-Event Analyses of the Walk On! Intervention on the Primary Composite Outcome of All-Cause Hospitalizations, Observation Stays, Emergency Department Visits, and Death from Months 2-12 Post-randomization

Supplement 3. **Data Sharing Statement**
