# Supplementary material for: Effect of Physical Activity Coaching on Acute Care and Survival Among Patients With Chronic Obstructive Pulmonary Disease: A Pragmatic Randomized Clinical Trial
Source: JAMA Netw Open. 2019 Aug 16;2(8):e199657. doi: 10.1001/jamanetworkopen.2019.9657 (PMC6704745; doi:10.1001/jamanetworkopen.2019.9657)
Supplement: Supplement 1. — Trial Protocol [file jamanetwopen-2-e199657-s001.pdf]

Walk On!

# PATIENT-CENTERED PHYSICAL ACTIVITY COACHING IN COPD: A PRAGMATIC TRIAL

## Study Protocol

Updated December 2018

# Table of Contents


EXECUTIVE SUMMARY ........ 3
BACKGROUND ........ 5
SPECIFIC AIMS ........ 5
STUDY DESIGN AND METHODS ........ 6
Study Setting ........ 6
Overview of Design ........ 6
Identifying Eligible Patients from the EMR ........ 8
Randomization ........ 10
Consent and Passive Monitoring ........ 11
Standard Care (SC) Control ........ 11
Rationale for Standard Care as a Comparator ........ 11
Study Procedures ........ 11
Walk On! Physical Activity Coaching Intervention ........ 12
Measures and Outcomes ........ 21
Analytical Plan ........ 24
Sample Size Estimates ........ 26
Safety Monitoring ........ 27
Project Milestones and Timeline ........ 27
REFERENCES ........ 28

# EXECUTIVE SUMMARY

**BACKGROUND**

Chronic obstructive pulmonary disease (COPD) is the third leading cause of the death in the US. The personal, social and economic costs of the disease are tremendous, with annual expenditures of nearly $50 billion, mostly from hospitalizations for exacerbations of COPD. For the vast majority of patients, despite optimal pharmacological therapy, living with COPD is characterized by unrelieved dyspnea, physical inactivity, deconditioning, and an insidious downward spiral of social isolation and depression. There is mounting epidemiological evidence that physical inactivity is associated with more frequent hospitalizations and increased mortality in COPD even after adjusting for disease severity. The evidence is unequivocal that intensive supervised exercise training as part of pulmonary rehabilitation improves outcomes of importance to patients. However, patient participation in supervised exercise at center-based rehabilitation programs is very low (1-3% of eligible patients) which undermines the wide scale adoption of this approach in real world clinical settings for large numbers of patients. A paradigm shift is needed in the non-pharmacological care of COPD from traditional rehabilitation to a more patient-centered, scalable, and sustainable model of promoting active lifestyles to improve outcomes for COPD and its common co-morbidities. Identifying alternative, more flexible models that honors patients' preferences and needs is of intense interest to patients and their caregivers.

**OBJECTIVES**

We propose a pragmatic randomized controlled trial in a large integrated health care system to determine the effectiveness of a 12-month patient-centered, physical activity coaching intervention (Walk On!) for patients with COPD on the primary composite outcome of all-cause hospitalizations, emergency department (ED) visits, observation stays and death compared to standard care. We will also examine the secondary outcomes of physical activity, symptom burden, quality of life, COPD-related health care utilization, and cardio-metabolic markers. The long-term objective is to scale-up and spread the implementation of this model into existing care management efforts across Kaiser Permanente and other health care systems should the findings be positive.

**METHODS**

A randomized controlled design was used to test the effectiveness of Walk On! compared to standard care (SC) in patients with a history of COPD-related hospitalization, ED or observation visits in the previous 12 months. Eligible patients (n=1650; revised to 2707) were automatically identified from our electronic medical records (EMR) system and randomized to either the Walk On! program or SC from July 2015-July 2017. SC patients continue to have access to all the health services they would receive such as a readmission reduction bundle, pulmonary rehabilitation, and health education programs. Walk On! patients will receive SC plus the individually tailored Walk On! program over 12 months which includes four components: baseline orientation/functional assessment, intensive coaching, and pro-active professional and peer support and monitoring via semi-automated outreach by telephone or Internet as well as group visits. Outcomes will be analyzed using conventional statistical methods for binary and continuous variables and according to intention to treat principles. We will also examine heterogeneity of effects in patient subgroups.

**PATIENT OUTCOMES**

Our primary composite outcome will be all-cause hospitalizations, emergency department (ED) visits, observation stays and death in the 12 months following enrollment in Walk On! Secondary outcomes include physical activity, COPD-related health care utilization, and cardio-metabolic markers such as

BMI, blood pressure, HbA1c, and lipids, all of which will be automatically captured from our EMR system; we will also measure patients' symptom burden, quality of life, perception of support, and satisfaction using mail, phone or web surveys. These outcomes were selected based on patients' expressed desires to remain independent for as long as possible in the face of a progressive illness.

**PATIENT AND STAKEHOLDER ENGAGEMENT**

The research team has engaged all relevant stakeholders including patients, family members, front line clinicians, administrators, and executive health system leadership from the proposal development stage to the planning/start-up, implementation, evaluation, and dissemination activities. A Patient and Family Advisory Board (PAB) met via telephone conference once a month to discuss study progress and partnered with the research team to address any study-related issues and challenges as they unfolded. In addition to the monthly calls, the PAB and health system partners met in person in Year 1 to inform the final study protocol, in Year 2 to refine the study processes and implementation, in Year 3 to celebrate the end of recruitment and plan for possible dissemination activities, and in Year 4 to review the study findings and strategize on next steps.

**ANTICIPATED IMPACT**

A pragmatic trial of physical activity coaching in high risk COPD patients is unprecedented. If successful, findings from this study could re-define the standard of care for patients with COPD to more aggressive management of physical inactivity in community and home-based settings in contrast to the current highly inaccessible gold standard center-based pulmonary rehabilitation programs. The Walk On! program could potentially provide patients and their families an effective alternative care model that meets their preferences and needs and payers will be able to invest in a more scalable intervention with more durable effects. Generating rigorous evidence regarding the impact of patient-centered behavioral interventions for individuals with multiple chronic conditions significantly advances PCORI's mission of assisting diverse stakeholders in making more informed decisions that reflect their desired health outcomes.

**PARTICIPATING KAISER PERMANENTE SOUTHERN CALIFORNIA MEDICAL CENTERS**

Downey, LAMC, Orange County, Riverside, Fontana, and San Diego (South Bay and West Los Angeles added in 2017)

**CORE INVESTIGATORS**

Huong Nguyen, PhD, RN (PI); Michael Gould, MD, MS; Karen Coleman, PhD; Smita Desai, DO; William Towner, MD; Anny Xiang, PhD; Marilyn Moy, MD, MS

**PATIENT and FAMILY ADVISORY BOARD**

Ms. Adrienne Bailey (deceased), Mr. Kenneth Desjardins (deceased), Ms. Leslie Paskus, Ms. Freida Miller, Ms. Reta Coulombe, Ms. Gloria Miller, Mr. Ronald Fox (deceased), Ms. Susan Barlett, and Ms. Bonnie Tomeoni

**DATA SAFETY MONITORING BOARD MEMBERS**

Kevin Cain, PhD, David Au, MD, MPH, Barbara Sternfeld, PhD

**REGULATORY**

KPSC Institutional Review Board Protocol: #10697
clinicaltrials.gov registration: #NCT02478359

# BACKGROUND

Chronic obstructive pulmonary disease (COPD) is the third leading cause of the death in the US.[1] The personal, social and economic costs of the disease are tremendous, with annual expenditures of nearly $50 billion, mostly from hospitalizations for exacerbations of COPD and associated sequelae.[2] For the vast majority of patients, despite optimal pharmacological therapy, living with COPD is characterized by unrelieved dyspnea, physical inactivity, deconditioning, and an insidious downward spiral of social isolation and depression that has a profound impact on the lives of patients and their caregivers.[3,4]

Physical inactivity is significantly associated with more frequent hospitalizations and increased mortality in COPD even after adjusting for disease severity.[5-10] Our recent findings further extend these observations by showing that hospitalized COPD patients who reported engaging in any level of moderate to vigorous exercise prior to the index admission had a 34% lower risk of 30-day readmission compared to inactive patients.[11] Reducing 30-day hospital readmissions has become a major focus of many health care systems, with most efforts targeted at addressing deficiencies in care transitions and short-term outpatient management following discharge.[12-14] Missing from many of these efforts is the recognition that a majority of hospitalizations for chronic illnesses like COPD reflect failures in aggressive and proactive outpatient management of COPD exacerbations, comorbidities, and behavioral risk factors.

The evidence is unequivocal that pulmonary rehabilitation (PR) improves symptoms, health-related quality of life, and exercise capacity in COPD.[15,16] While PR has been a guideline-recommended therapy since 2001and is a Medicare covered benefit, uptake remains suboptimal, with only 1-3% of eligible patients ever participating.[17,18] The organizational, provider, and patient-level barriers to participation are well-known, persistent, and remain inadequately addressed.[17-19] Moreover, gains from PR have a predictable decay over 6-12 months since most patients are not able to sustain these lifestyle changes independently in the face of a chronic progressive illness.[15,20-22] Novel approaches that overcome limitations of the current model are desperately needed in order to achieve the triple aim of more patient-centered care, with better outcomes at lower cost, for the vast majority of patients who currently cannot access PR.

There is significant recent interest and focus on testing lifestyle PA interventions.[23,24] Several different PA intervention models have been tested by us and others that combine various elements of supervised[25,26] and independent exercise[27], some with the use of pedometers[28-31] alone or in combination with a motivational Internet-enabled platform[32-34] and telephone based coaching.[26,28,31,32] Together, findings from these published studies suggest that patients with COPD can increase their PA. Despite the growing evidence base for PA interventions, there is a critical gap regarding the real-world effectiveness of improving PA in large representative samples of older adults with COPD and its impact on widely accepted clinical endpoints.

# SPECIFIC AIMS

1. Refine a patient-centered physical activity coaching (Walk On!) intervention model to improve outcomes for patients with COPD who are at high risk for hospitalization.
2. Conduct a pragmatic randomized controlled trial to determine the effectiveness of the Walk On! intervention compared to standard care on the primary composite outcome of all-cause hospitalizations, observation stays, emergency department visits, and mortality and secondary outcomes of COPD-related hospitalizations, observation stays and emergency department visits,

number of outpatient treated COPD exacerbations, physical activity, cardio-metabolic markers, symptoms, and health-related quality of life.

Hypothesis: Patients randomized to Walk On! will have a 7% (revised: 5.5%) absolute reduction in the primary composite outcome in the 12 months after randomization compared to standard care patients. Walk On! patients will also have increased physical activity, fewer COPD-related encounters, better cardio-metabolic markers, lower symptoms, and improved quality of life.

3. Examine the effectiveness of Walk On! in patient subgroups (presence of multi morbidities, level of social support, gender, race/ethnicity, and access to the Internet).
4. Use mixed methods to understand the barriers and facilitators of successful uptake of the Walk On! intervention components.

# STUDY DESIGN AND METHODS

## Study Setting

Kaiser Permanente Southern California (KPSC) is a large integrated health care system that provides comprehensive health care services for approximately 1 in 5 Southern California residents (~4.5 million members). Kaiser Permanente is the largest real-world care setting in the nation and the ideal test-bed to conduct cost-effective pragmatic trials of innovative lifestyle behavioral models. Members enroll through the Kaiser Foundation Health Plan for prepaid health care insurance. KPSC provides care at 14 hospitals, 16 medical service areas, and nearly 200 medical offices through a partnership of more than 6,000 physicians. There is a robust and comprehensive electronic medical record system (Epic™) and online patient health portal (kp.org).

## Overview of Design

A conceptual model guiding this study is illustrated in Figure 1. We hypothesize that increased physical activity leads to improvements in both physiological (decreased ventilatory requirements and breathlessness, improved cardio-metabolic management, and lower levels of inflammation) and psychological (improved mood, lower anxiety and increased self-efficacy for self-care) factors. Changes in these mediators are associated with increased quality of life, fewer COPD exacerbations, decreased acute care utilization, and improved survival.

Figure 1. Walk On! Conceptual Model

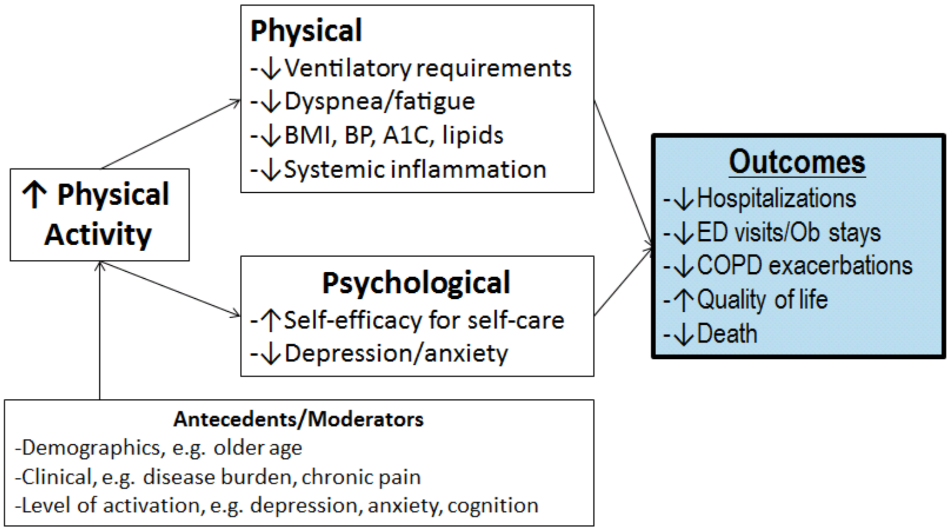

This study is a pragmatic randomized controlled trial to determine the effectiveness of a physical activity coaching intervention (Walk On!) compared to standard care for patients at high risk for COPD exacerbations (Figure 2). Our research question, study design, and proposed methods are aligned with methodological standards for pragmatic or real-world clinical trials (Table 1).[35] We used automated methods to identify approximately 1,650 (revised to 2,707) eligible patients with a COPD-related hospitalization, emergency department visit, or overnight observation stay in the previous 12 months from KPSC electronic medical records system (EMR) and randomized patients in a 1:1 ratio to the Walk On! intervention or standard care. Patients randomized to Walk On! were approached by existing KPSC clinical staff (respiratory therapists who served as physical activity coaches) to participate in the 12-month physical activity coaching program. Patients randomized to standard care were not contacted about the trial with the exception of a subgroup (n=250, revised to 537) who were invited to complete surveys over 12 months. Patients were enrolled in waves over 24 months.

The primary outcome was a composite of all-cause hospitalizations, emergency department visits, observation stays, and death in the 12 months following randomization. Secondary outcomes included COPD-related encounters, cardio-metabolic markers (BMI, BP, HbA1C, and lipids), self-reported physical activity, symptoms, health-related quality of life (HRQL), perception of support for PA, and satisfaction with the program. All utilization and clinical outcomes were captured from the EMR. Patient reported outcomes (PROs) were measured with standardized surveys.

Table 1. PRECIS Pragmatic Study Design

| **PRECIS Criteria** | **Criteria for Pragmatic Trials** | **Physical Activity Coaching Trial (Walk On!)** |
|---|---|---|
| Participants | All eligible participants enrolled, regardless of risk, responsiveness, comorbidities or past compliance. | All adult health plan members who had a COPD hospitalization/ED visit/Ob Stay in the past 12 months (excluding patients who clearly would not benefit from Walk On) were enrolled. |
| Intervention Condition | Interventions are highly flexible, offering providers leeway in formulation and application. | Walk On! allowed for tailoring to patients' needs and preferences. Varying levels of participation were expected. |
| Intervention Practitioners | Interventions are applied by the full range of practitioners in the full range of settings with only ordinary attention to dose and side effects. | Intervention clinicians were recruited from the existing local workforce (respiratory therapists). The sites were responsible for selection and supervision of clinicians (using standard quality control tools). |
| Comparison Condition | "Usual Practice" (or the best alternative), offering practitioners considerable leeway in application. | Walk On! was compared to standard of care that members with COPD receive based on their existing health plan benefits. |
| Comparison Practitioners | The control intervention is applied by the full range of clinicians in the full range of settings, with only ordinary attention to training, experience, and performance. | Standard care was provided by real-world providers under usual practice conditions – with no additional training or supervision. |
| Follow-Up Assessments | There are no research assessments; existing databases are searched for outcomes. | All utilization and clinical data were collected from existing electronic medical records and insurance claims data. Limited patient-reported outcomes were collected from intervention patients and a randomly selected subgroup of standard care patients |
| Outcome Definition | The primary outcome is objectively measured, meaningful to study participants, and does not depend on central adjudication. | Primary and secondary outcomes were defined by utilization, pharmacy, and clinical data (exercise; cardio-metabolic markers). No additional clinical assessment was required. |
| Intervention Compliance | There are no special strategies to improve compliance, and compliance is unobtrusively measured. | Quality of implementation were assessed using a study dashboard. |

| | | |
|---|---|---|
| Practitioner Adherence | There are no special strategies to maintain practitioner adherence, and adherence is unobtrusively measured. | Clinical supervision of physical activity coaches are at the same intensity as any operational clinical programs. |
| Primary Comparison | The analysis includes all patients regardless of compliance, eligibility, or others. | All outcomes will be analyzed according to initial assignment – regardless of intervention participation or compliance. |

Figure 2. Walk On! Study Design

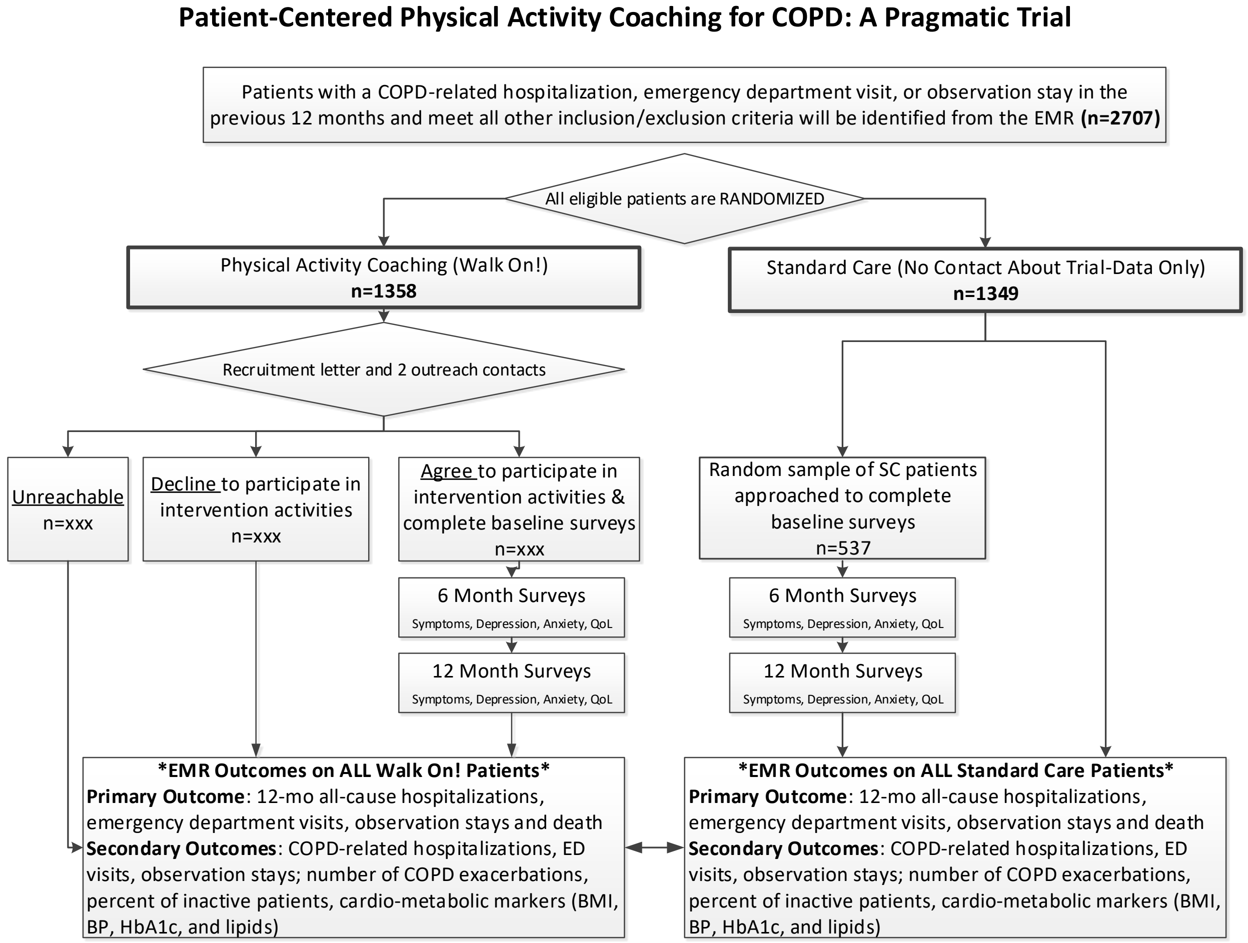


## Identifying Eligible Patients from the EMR

Eligible patients from six KPSC medical service areas were identified, randomized and enrolled in this study from July 2015-July 2017. The target sample size of 1,650 was increased to 2,700 in July 2016 due to the lower than expected uptake rate.

Inclusion criteria:

a) Patients with any COPD-related hospitalization, emergency department visit or observation stay in the previous 12 months are eligible for the study (June 1, 2014-present) from DO, OC, LAMC, RV, SD, and FO medical center areas (PARFU, previous utilization per MCA). Patients from WLA and SB who lived within 10 miles of DO and LAMC were sampled starting with Wave #10 to ensure we reached our new sample target.

(Original up to 9/31/15) COPD-related hospital-based utilization are defined according to the Centers for Medicare and Medicaid Services (CMS) and National Quality Forum (NQF) criteria for the Hospital Readmission Reduction Program. The following principal discharge diagnoses of COPD will be included (ICD-9 codes: 491.21, 491.22, 491.8, 491.9, 492.8, 493.20, 493.21, 493.22, and 496) or respiratory failure (ICD-9 codes: 518.81, 518.82, 518.84, 799.1) with a secondary diagnosis of COPD exacerbation (ICD-9 codes: 491.21, 491.22, 493.21, 493.22)

Updated Effective 10/1/15: Any COPD-related hospitalization, emergency department visit or observation stay in the previous 12 months with principal discharge diagnoses of COPD (J44.1, J44.0, J41.8, J42, J43.1, J43.2, J43.8, J43.9, J44.9, J44.0, or J44.1) or principal diagnosis of respiratory failure (J96.00, J96.01, J96.02, J96.90, J96.91, J96.92, J80, J96.20, J96.21, J96.22, or R09.2) and a secondary diagnosis of acute exacerbation of COPD (J44.1 or J44.0)

Note: If patient has an external KP encounter, two COPD diagnosis codes are required separated by 2 days in order to be included to avoid miscoding which can occur frequently w/claims data.

| **ICD-9 Codes** (6/1/14-9/31/15) | **ICD-10 Codes** (10/1/15-Present) |
|---|---|
| **Principal discharge diagnosis** | |
| 491.21 | J44.1 |
| 491.22 | J44.0 |
| 491.8 | J41.8 |
| 491.9 | J42 |
| 492.8 | J43.1, J43.2, J43.8, J43.9 |
| 493.20 | J44.9 |
| 493.21 | J44.0 |
| 493.22 | J44.1 |
| 496 | J44.9 |
| 518.81+ either: 491.21, 491.22, 493.21, or 493.22 | J96.00, J96.01, J96.02, J96.90, J96.91, J96.92 + either J44.1 or J44.0 |
| 518.82 + either: 491.21, 491.22, 493.21, or 493.22 | J80 + either J44.1 or J44.0 |
| 518.84 + either: 491.21, 491.22, 493.21, or 493.22 | J96.20, J96.21, J96.22 + either J44.1 or J44.0 |
| 799.1 + either: 491.21, 491.22, 493.21, or 493.22 | R09.2 + either J44.1 or J44.0 |

b) Age >40 years at the time of the hospitalization/ED visit/Ob stay
c) On at least a bronchodilator or steroid inhaler prior to the encounter or if not on an inhaler, had a previous COPD diagnosis (any outpatient diagnosis is acceptable; inpatient diagnosis is acceptable only if it is a KFH admission)
d) Continuous health plan membership in the 12 months prior to cohort identification.

Exclusion criteria:

a) For patients with spirometry data, FEV1/FVC ratio >0.70 at any point in the 24 months prior to cohort identification; include FEV1/FVC pre-bronchodilator use if post value is unavailable
b) Discharged to hospice (look for hospice encounter in past 6 months up to cohort identification; add home-based PC service as exclusion due to homebound status), a skilled nursing facility, long term-care or another acute care hospital during the index admission. ***If patients are missing a discharge status (this is most common with non-KP encounters), they are excluded.
c) Level of function at admission or discharge is bed bound during the index admission
d) Has Alzheimers disease/dementia or metastatic cancer

| **ICD-9** | **ICD-10** (10/1/15-Present) |
|---|---|
| **Dementia/Alzheimers** | |
| 290, 290.10, 290.11, 290.12, 290.20, 290.21, 290.3, 290.40, 290.41, 290.42, 290.43, 290.8, 290.9 | F03.90, F05, F01.50, F01.51, |
| 294.10, 294.11, 294.20, 294.21 | F02.80, F02.81, F03.91 |
| 331.0, 331.11, 331.19, 331.82, 331.83, | G30.0, G30.1, G30.8, G30.9, G31.01, G31.09, G31.83, |

| | |
|---|---|
| | G31.84 |
| 780.93 | R41.1, R41.2, R41.3 |
| **Metastatic/Advanced Cancers** | |
| 153.9 | C18.9 |
| 162.9 | C34.90, C34.91, C34.92 |
| 174.9 | C50.911, C50.912, C50.919 |
| 183 | C56.1, C56.2, C56.9, C57.00, C57.01, C57.02, C57.10, C57.11, C57.12, C57.3, C57.20, C57.21, C57.22, C57.4 |
| 196 | C77.0, C77.1, C77.2, C77.3, C77.4, C77.5, C77.8, C77.9 |
| 197 | C78.00, C78.01, C78.02, C78.1, C78.2, C78.30, C78.39, C78.4, C78.5, C78.6, C78.7, C78.80, C78.89 |
| 198 | C79.00, C79.01, C79.02, C79.10, C79.11, C79.19, C79.2, C79.31, C79.32, C79.40, C79.49, C79.51, C79.52, C79.60, C79.61, C79.62, C79.70, C79.71, C79.72, C79.81, C79.82, C79.89, C79.9 |

e) Morbidly obese (BMI >40) at time of cohort identification
f) Completed pulmonary rehabilitation in the 6 months prior to cohort identification (OVG150 visit code for internal PR; G0424, G0237, 97150, 97530, 97110 for claims data with duration of at least 30 days between the first and last session for all claims)
g) Deceased at cohort identification
*h)* Dis-enrolled from the health plan at cohort identification

## Randomization

We considered and rejected the option of group- or cluster-level randomization. The core Walk On! components were applied at the level of the individual patient rather than the provider or clinic, so cross-over or spill-over of intervention effects within clinics or providers should not occur. Consequently, there was no scientific advantage to cluster-level randomization.

All eligible patients were randomly assigned 1:1 to Walk On! or to continue with standard care, stratified by medical center, time from hospitalization/ED/Ob Stay (<6months vs. ≥ 6months), level of physical activity obtained from the exercise vital sign closest to the cohort identification date (inactive: 0 min/week of moderate/vigorous physical activity, MVPA vs. active: at least 1 min/week of MVPA; patient will be assumed to be inactive if no EVS available), and median age (<72 vs. ≥72) by random permuted blocks to ensure balance and reduce bias. The randomization was pre-generated and included two steps for each stratum. First, the block size was randomly selected among a set of pre-selected block. Second, within each block randomly selected, the overall number of treatment assignments was balanced between groups, but the order of treatment assignment was randomly assigned. We repeated this process until the number of maximum expected patients was reached. We reviewed every 6 months to determine balance across the two groups on these characteristics and did not make any modifications to the scheme.

For standard care patients (n=250; revised to n=537 due to a lower than expected response rate) who were approached to complete surveys, with each recruitment wave, we randomly selected patients from the six sites proportional to the number of patients randomized to Walk On! We sent surveys to all SC patients on the final wave due to the low response rate from the earlier waves.

The anchor date for all patients is the date they are identified and randomized to treatment arms. For example, a patient had a COPD admission/ED/Obs encounter on June 25, 2014 and is identified for the study on June 30, 2015, the anchor date would be June 30, 2015. The 12-month pre-

intervention period would include the index encounter (June 25, 2014 to June 30, 2015) and the post-intervention period would be July 1, 2015 to June 30, 2016.

## Consent and Passive Monitoring

Following a single consent design,[36,37] only those assigned to Walk On! were asked to consent to intervention activities. Patients assigned to standard care were not contacted about the study. Our IRB considered passive monitoring of outcomes using existing EMR data a minimal risk activity that did not require patient consent. We believe that such an approach is both scientifically necessary and ethically justified. Acceptability of Walk On! to patients and level of participation are essential components of real-world effectiveness. If we limited trial enrollment to those who volunteer to receive Walk On!, findings regarding intervention acceptability or adherence may have limited scientific value. Moreover, we believed the subset of patients who agree to be in an RCT is substantially different from the subset of patients who would agree to do Walk On! if offered it; findings regarding intervention effects on the primary outcome would have questionable validity and limited generalizability.

## Standard Care (SC) Control

Standard care patients continued to receive their routine care from KPSC and had access to all health services, e.g. primary, specialty care, pulmonary rehabilitation, health education and lifestyle programs in accordance with their health plan. Standard care patients received no instructions to exercise and were not contacted about the trial, with the exception of a randomly selected subgroup (n=250, revised to 537) with each recruitment wave to complete PROs at 6 and 12 months (with exception of satisfaction surveys) for comparison to the Walk On! patients.

## Rationale for Standard Care as a Comparator

We chose to compare the Walk On! intervention to standard of care for the following reasons: 1) there were insufficient data from large scale studies to support any specific PA intervention model for COPD and most small efficacy studies have had restrictive inclusion criteria; and 2) since there are no scalable programs available, standard care (which includes access to pulmonary rehabilitation) was the most appropriate comparator from the perspective of the healthcare system that is considering implementing the intervention and the individual patient who is considering participating in the intervention. We considered an active control group with mail outreach to remind members of the programs available to them. However, this would not increase the scientific value of the study, given that such low intensity touches are historically known to be ineffective.

## Study Procedures

For those patients randomized to Walk On!, a recruitment packet that included a letter signed by the principal investigator and the pulmonary physician in charge for the medical service area, a study brochure describing the Walk On! program, and a 3-min DVD video “testimonial” (http://abc7.com/archive/9501102/) about the importance of PA by one of the Patient Advisory Board (PAB) members was mailed within 1-6 business days of cohort selection and randomization. (Note: We stopped mailing the DVD after wave 4 due to patient and coaches’ feedback that they were not being watched. Uptake of Walk On! was not negatively impacted by eliminating the DVD from our recruitment packet.) Patients had the option of calling the physical activity coach to either agree to participate, or to opt-out in response to the mailing. If patients did not actively call the PA coach for more information or to opt-in to the study, the coaches conducted a total of two outreach contacts via phone and/or secure message seven business days after the mailing. When contact was made with the patient, the coach described the purpose of the study, how the patient was selected for participation, the Walk On!

intervention, and time commitment. After addressing the patient's questions, the coach obtained oral consent and scheduled the baseline orientation intake visit. Patients did not have any further contact to recruit them once the recruitment packet was sent and two contact attempts were made.

Patients who agreed to participate in Walk On! activities were sent a baseline packet approximately ten days before their scheduled baseline visit that included: 1) a consent form, 2) an activity sensor, 3) an additional copy of the study brochure, and 4) surveys to assess their physical activity, symptom burden (COPD Assessment Test, CAT), depression (PHQ-8), and anxiety (GAD-7), and quality of life (PROMIS-10). Patients were asked to wear one of two available sensors for up to seven days prior to the baseline visit. After the visit, patients received four weekly coaching phone calls. Outreach by the PA coaches for the remaining 11 months were individualized and targeted based on patients' progress with their walking program. Patients were also encouraged to attend monthly peer support meetings.

At six and 12 months after their randomization date, patients were sent a survey packet and a $5 gift card. A reminder letter was sent if the surveys are not received within two weeks. Finally, a phone follow-up was made seven business days after the reminder letter. Patients had the option of providing their survey responses over the phone.

For standard care patients, a random sample (n=250, revised to 537) was invited to complete the same set of surveys at baseline, six and 12 months, with the exception of the satisfaction questions, in order to compare changes in PROs between intervention and standard care patients. These patients were only informed that their medical center is participating in a study to improve outcomes for members with COPD. We used the same mailing and phone-based follow up procedures described above.

## Walk On! Physical Activity Coaching Intervention

***Theoretical Foundations***

The Walk On! intervention was designed based on learnings from a series of collective studies[45,47,48,85] by the investigative team that were informed by early and deep engagement with patient stakeholders and is grounded in social cognitive[38] and self-regulation theories[39,40] and core principles of motivational interviewing (Table 2).[41] In self-efficacy theory, the impetus for change resides in the individual's efficacy expectations or one's "confidence in one's ability to take and persist in action." These expectations reflect a person's beliefs about how capable he or she is in performing a task. External environmental supports, like professional, peer and family modeling and engagement in similar behaviors also increases efficacy. Walk On! had three core components (baseline assessment/orientation, intensive coaching, and pro-active support) with built in flexibility to accommodate the diverse preferences and needs of patients as well as anticipated implementation constraints. We focused on promoting walking as the primary mode of PA since nearly 90% of activities that patients with COPD engage in are ambulatory in nature and it is a safe and accessible form of PA.[42] The estimated time commitment ranged from 6-18 hours over the course of 12 months depending on patients' participation in various Walk On! activities.

**Table 2. Walk On! Intervention Mapping**

| Target Concept (Source) | Walk On! Strategy |
|---|---|
| Enhancing self-efficacy for increasing physical activity | |
| • Performance or enactive accomplishments | Guiding patients to set achievable walking goals each week to increase their mastery of walking gradually |
| • Re-interpretation of signs and symptoms | Practicing breathing strategies to cope with dyspnea during walking, and, over time, developing a recognition that one can do more with the same level of dyspnea (desensitization to dyspnea)<br>Tracking and reporting symptoms every week increased awareness of changes in symptoms that might interfere with walking and daily activities to facilitate earlier treatment and reduce disease-specific barriers to walking |
| • Vicarious experience | Social modeling allowed patients to be positively influenced by the achievement of other participants initially during the orientation session and during the ongoing monthly group meetings |
| • Social persuasion | Encouragement from coaches and peers during orientation session; ongoing reinforcement from coaches via phone/secure messaging; and peer interactions during monthly meetings |
| Exercise specific social support | Identification of family or friends to support efforts at increasing physical activity, including attendance at Walk On! activities<br>Peer support and networking during monthly group meetings that include enactment of walking/light exercise |
| Iterative rational behavior change | |
| • Accurate self-monitoring | Study-issued step counting devices allowed patients to track their daily progress accurately |
| • Incremental goal setting | Dynamic individualized incremental goals suggested by the IVR and Internet-based intervention platforms |
| • Motivational feedback | Patients received real-time feedback from the step counting devices and personalized motivational feedback and guidance as needed from the coaches |

**Walk On! Intervention Components**

***A. In-person individual or group orientation visit (Week 0)*** Patients had the option of attending the orientation session individually or with one other patient who lived within a close geographical area to promote peer bonding and support. Since social support[43] has been shown to be critical for behavior change, patients were also encouraged to identify and invite a family or friend care partner to this visit.

*1) Education and skills training on PA & COPD management*. During the visit, the coach discussed the importance of PA for COPD self-care, what patients hoped to achieve with increasing PA, how to manage their symptoms with PA, maintaining safety with PA, and strategies to overcome personal barriers to regular PA. Patients were provided a paper copy of the Walk On! Patient Guide (Appendix Section 2).[30]

*2) Baseline functional assessment for PA prescription*. Patients completed a six-minute walk test (6MWT) while wearing their activity sensor and had their oxygen saturation and heart rate measured pre- and post- test. Patients who desaturated <88% at the end of the 6MWT were evaluated for supplemental oxygen prior to starting their PA program. The coach (see detailed description below) tailored the initial walking prescription according to patients' performance on the 6MWT and their average steps/day during the baseline 7-day monitoring period; they used the higher of the two step counts as an initial step goal. Our previous data showed that patients typically perform at approximately 60% of the walking intensity achieved during the 6MWT.[44] Thus, we derived a step count goal of total steps accrued during the 6MWT multiplied by a factor of 5 to achieve approximately 30 minutes of

walking per day. For instance, a patient who accrued 500 steps during the 6MWT might be asked to aim for 1500 steps/day during week one (500 x 5 x 0.60 = 1500 steps/day). For patients who were more frail and for whom walking would initially be difficult due to severe deconditioning, we loaned a portable cycle ergometer for patients to use during the first four weeks to strengthen their walking muscles and gradually progressed them to a walking program.

*3) Training on use of activity sensors & resistance bands*. Patients chose one of two devices to monitor their step counts, the Omron HJ329 pedometer or Tractivity accelerometer based on their preference and access to the Internet, and were trained on their proper use. The Omron has an on-device display whereas the Tractivity device displays step count data via any Internet or Blue-tooth enabled device. Patients who did not have Internet access or were Spanish speakers were encouraged to use the Omron pedometer since they would be able to see their step counts more easily and could report their average weekly step counts to our automated telephone interactive voice response (IVR) system (Spanish script), respectively. The Omron pedometer was worn on the waist and had been validated and used in several of our COPD studies.[30,45] (Appendix Section 2 )

The Tractivity, worn on the ankle, was validated against a research grade accelerometer in a general population of hospitalized medical-surgical patients (n=20)[46] and used by patients in our pilot with acceptable concordance with a research grade accelerometer (Stepwatch). Patients who chose to use Tractivity were shown how to download a small applet on their Internet-enabled device and how to view their step counts. (Appendix Section 2)

Note: The vendor that provided the Tractivity sensors went out of business in December 2016. We were only able to offer patients the Omron device with recruitment waves 10 and 11 while we worked on evaluating alternative devices, selecting and testing our top selection and configuring and testing our systems to accommodate a new device for the final wave. We were able to offer patients the option of using a wrist-worn, FitBit Alta or an Omron device for wave 12. We also converted patients from the earlier waves who had challenges with using their Omron device to the FitBit if their coaches felt that having the FitBit device help with engagement and motivation. (Appendix Section 2)

Since breathlessness with daily activities that involve the upper extremities is common in this population, patients were also instructed on arm exercises using study-issued resistance bands to strengthen their upper extremities. They were asked to complete these arm exercises 3 times/week, but these exercises were not closely tracked.

***B. Intensive coaching (Weeks 1-4).*** We have found that the initial weeks of starting a walking program are most critical and are a time when patients require significant support to solve problems and barriers that arise as they integrate a new activity in their daily lives. Thus, the coach conducted weekly phone calls to help patients progress with their PA goals, reinforce COPD self-care skills, support patients' efforts to monitor their activities and symptoms, assist with problem solving PA barriers, and troubleshoot any device or technology issues. The coaches were guided by key principles of motivational interviewing such as expressing empathy, rolling with resistance, and supporting self-efficacy[41] to personalize the content of these calls according to the patients' progress. The coaches made appropriate referrals to either the patient's primary care provider or pulmonologist regarding any clinical issues that needed to be followed up on. Patients were closely guided on how to safely resume their PA after experiencing a COPD exacerbation. In addition, participants were instructed during the baseline orientation to know when to stop their PA and seek emergent care (e.g. significant increases in their dyspnea, chest pain or tightness, or other severe pain associated with activity).

***C. Proactive follow-up and support (Weeks 5-52).*** Development of a new habit such as PA requires regular practice, collaborative monitoring, and ongoing reinforcement and support from credible peer models. Regardless of which activity sensor patients used, both systems were designed to support dynamic, timely feedback, and individualized, iterative goal setting.

*1) Proactive monitoring and follow up.* Patients who used the Omron received an automated IVR phone call each week that queried them about their breathing, presence of any health issue(s) that interfered with their PA, and their average step count in the past week. Based on the patients' responses, a step goal was suggested for the subsequent week. These calls lasted, at most, 3 minutes. We recognized from our previous studies and feedback from our PAB that not all patients would agree to wear a pedometer to track their step counts; thus, we built into our IVR system an option for patients to enter the frequency and duration of their PA. The IVR system provided recommendations for PA duration instead of steps in these situations.

Patients who used the Tractivity device transmitted their data to an Internet-enabled device (smartphone, tablet or laptop) via Bluetooth and responded to the same two questions about their breathing and health status as asked of Omron users, which generated the step goal recommendation for the subsequent week. Patients were encouraged to review the graphical summary of their step counts, which displayed past step data and suggested step goals.

The personalized step goal algorithm (Figure 4) was designed to ensure that the step progression was safe and minimized common adverse events such as increased muscle soreness, more dyspnea and fatigue associated with increasing PA. Email alerts were generated to the coaches when patients reported worsening breathing and health problems interfering with their PA.

Patients who used the FitBit Alta are not asked the weekly health and breathing questions due to our inability to deploy these questions within the FitBit web application nor were we able automatically generate suggested step goals based on their previous week's performance and survey responses. However, patients have access to all the various tracking functionalities available on the FitBit website to use at their discretion.

Figure 3. Walk On! Program

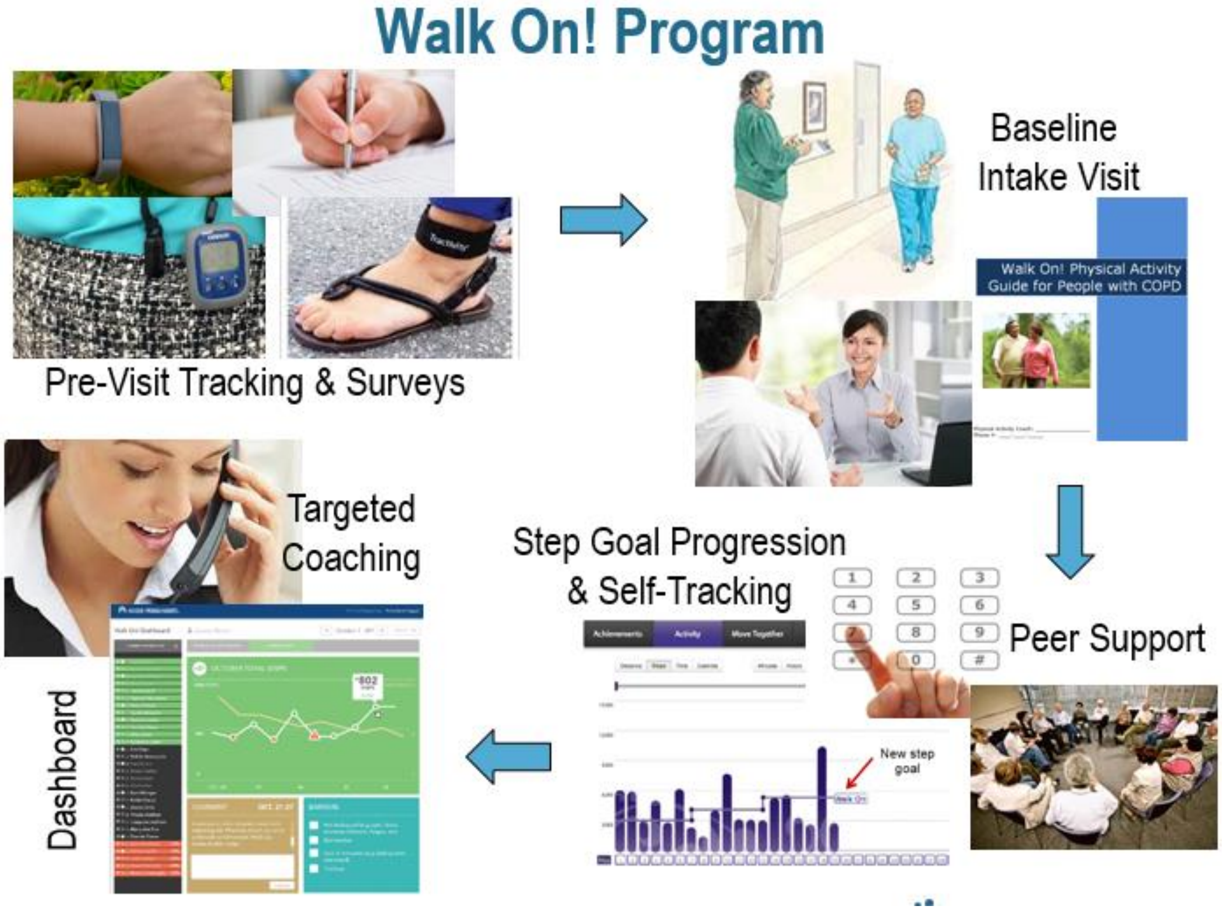

Figure 4. Step Goal Algorithm

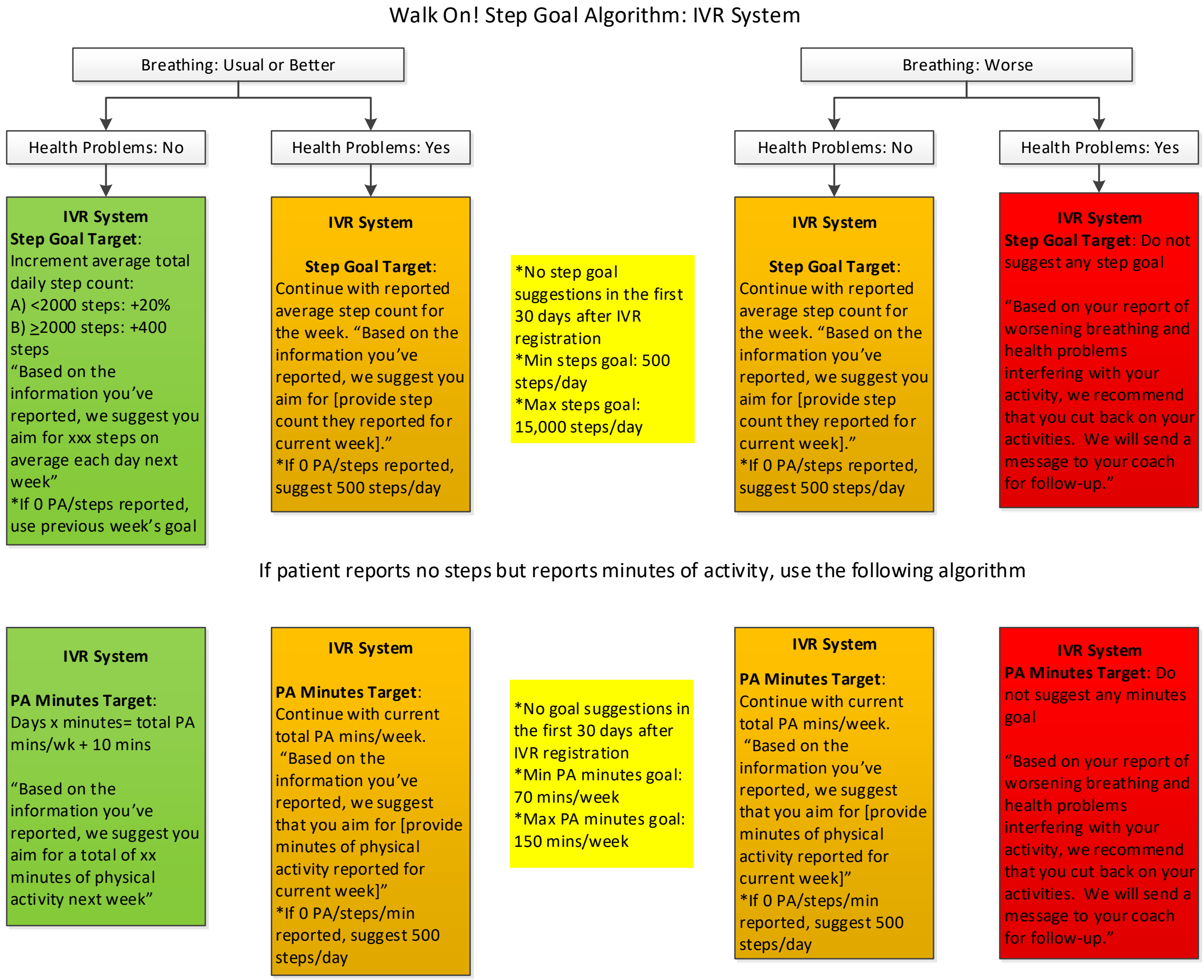


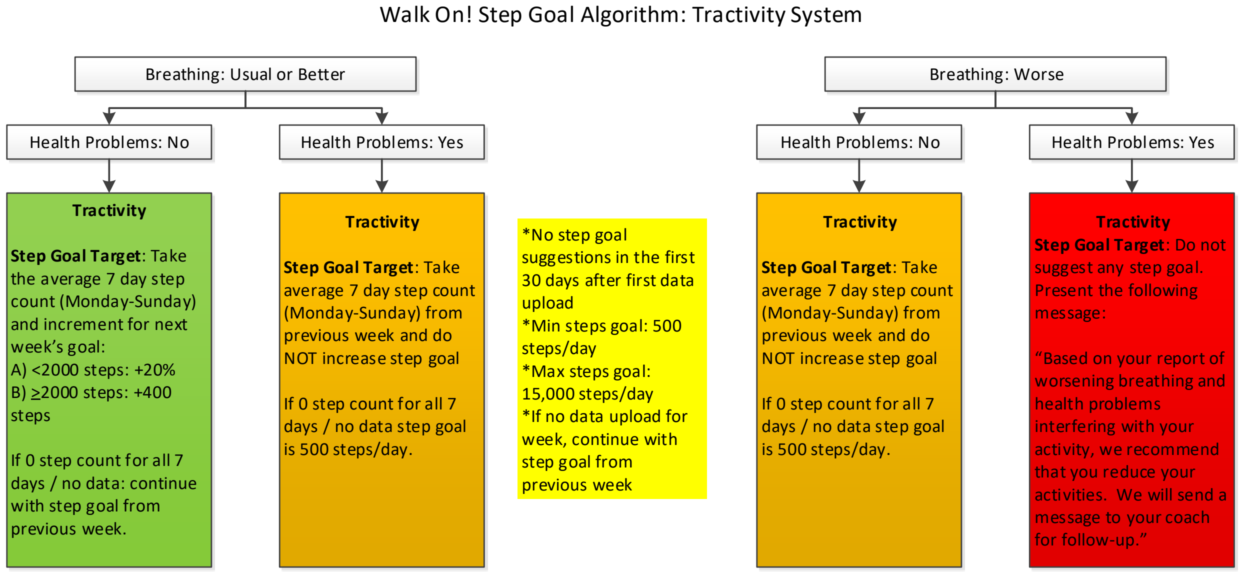

Data from both the IVR system and Tractivity web site were automatically retrieved and displayed on a dashboard for regular review by the coaches (Figure 4). The dashboard facilitated population management and targeted phone/secure message outreach to patients who were struggling to progress with their walking goals and/or had more severe symptoms than usual, in which case the coach communicated with the patient's provider as needed. The dashboard facilitated contact and workflow management, standardized documentation to track intervention exposure and thus increased the efficiency of the quality control/process evaluation efforts.

*2) Monthly group visits for psychosocial support from peers, skill-building, and problem solving*. Patients had the option of attending monthly hour-long support sessions with their family member or friend. These group visits started with 15-minutes of light exercise followed by 15-minutes of informal peer interactions and networking. Peer support is especially important for patients who feel they have limited support from their families. The meetings concluded with a 25-minute didactic/skill-building component that was broadcasted via the web and tele-conference.

The session topics focused on practical strategies to overcome common barriers to staying active, e.g. COPD exacerbations, weather, motivation and other relevant topics related to COPD management. The coaches collaborated in creating power point slides for these topics and collectively reviewed and approved the content for 12 topics. Other slide sets were developed on new topics that were either suggested by patients or nominated by the coaches.

Our PAB members participated in these sessions as their time allowed and, along with other peers, shared their successes with using community-based resources to stay active. Patients were entered into a raffle for a $20 gift card at each monthly meeting. Sites that have an active pulmonary rehabilitation program were encouraged to combine the Walk On group education visits with the rehabilitation education sessions to increase sustainability and efficiency in early to mid-2017. Two sites were successful in doing this mostly because they did not have a physical space constraint.

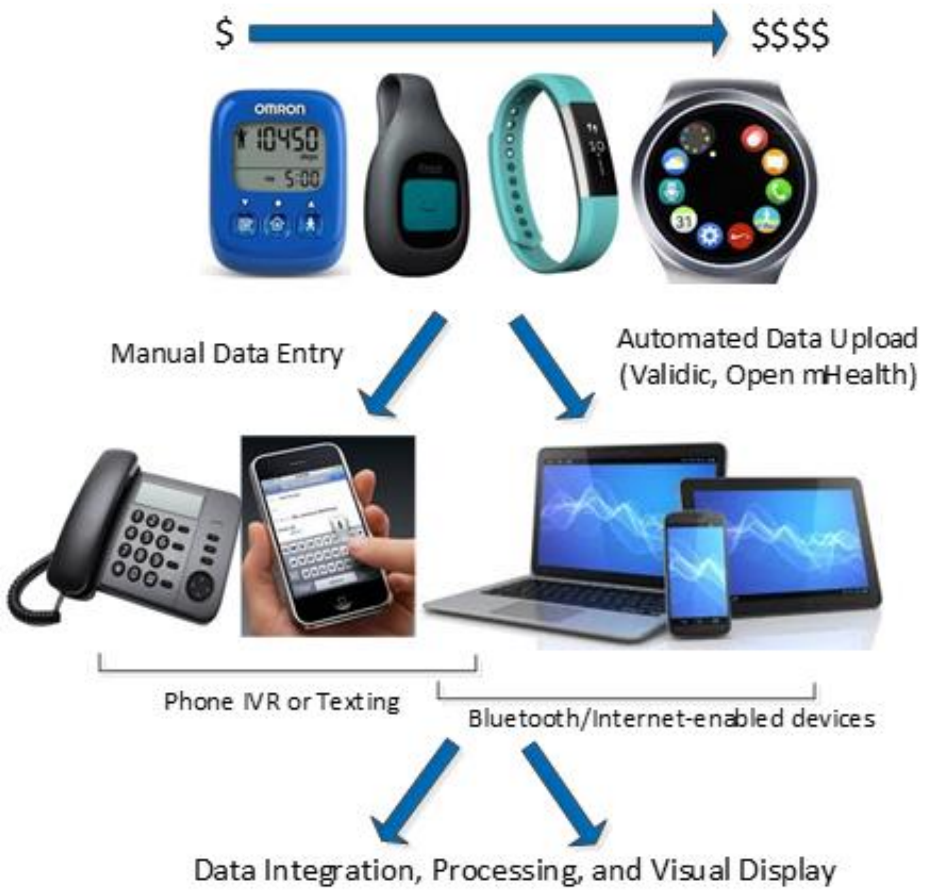


Figure 4. Coaches Dashboard (Note: Patient names are fictitious)

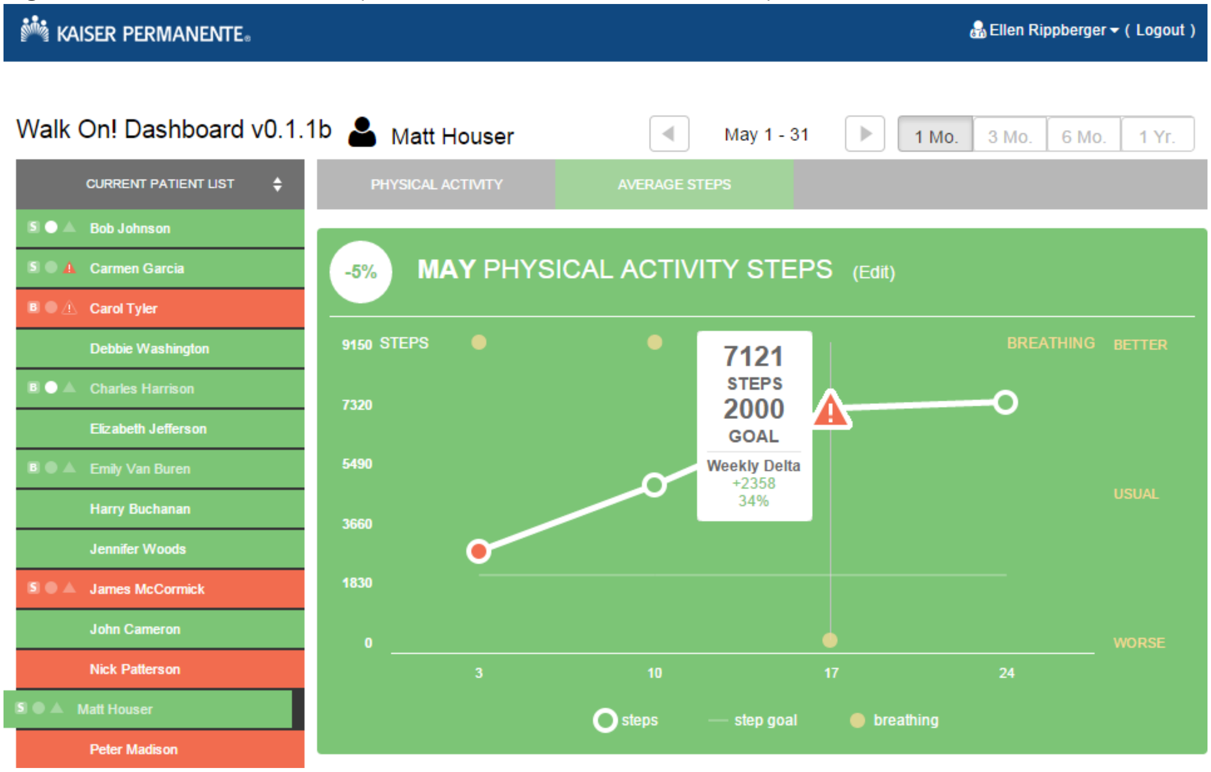

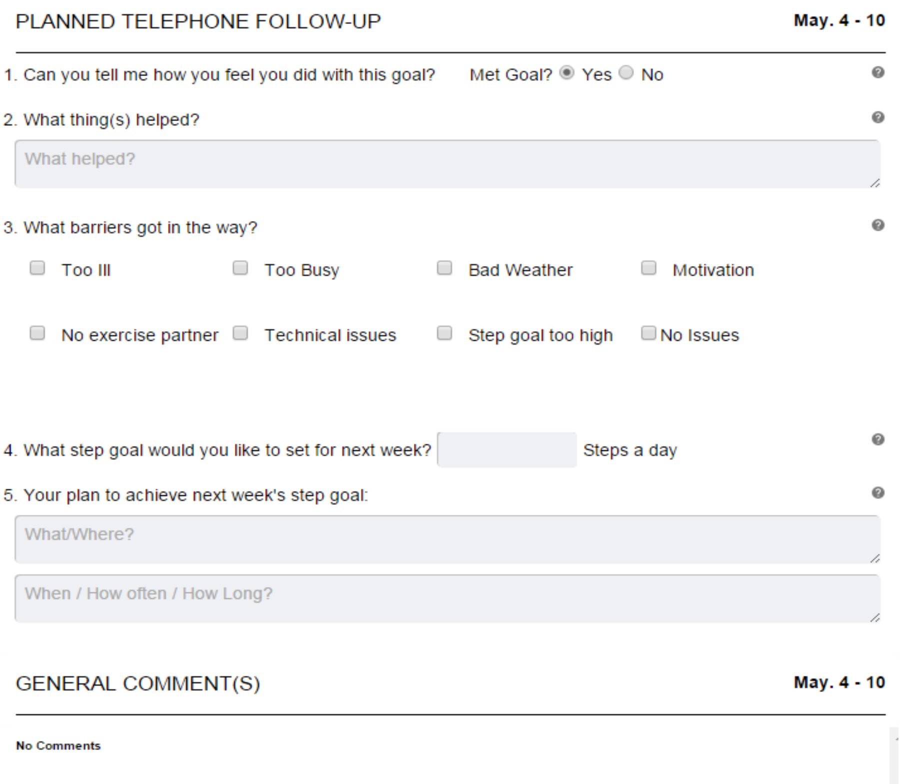

PLANNED TELEPHONE FOLLOW-UP May. 4 - 10

1. Can you tell me how you feel you did with this goal? Met Goal? Yes No

2. What thing(s) helped?

What helped?

3. What barriers got in the way?

Too Ill | Too Busy | Bad Weather | Motivation

No exercise partner | Technical issues | Step goal too high | No Issues

4. What step goal would you like to set for next week? Steps a day

5. Your plan to achieve next week's step goal:

What/Where?

When / How often / How Long?

GENERAL COMMENT(S) May. 4 - 10

No Comments

***Walk On! physical activity coach training***

Walk On! coaches were recruited from the existing KPSC workforce of respiratory therapists, pulmonary rehabilitation coordinators, and pulmonary care managers. The coaches participated in a general motivational training workshop offered to all health care providers in our system and a half day in-person project-specific training during the 6-month pilot phase prior to the study launch. The coaches were also provided a detailed guide of the Walk On! program. Each coach implemented the Walk On! protocol with 1-4 pilot patients for 3 months in preparation for the trial; the principal investigator (HQN) or one of the lead PA coaches observed and provided feedback to the coaches during their first 1-2 baseline visits. Issues or concerns with the phone coaching calls were discussed during weekly to bi-weekly web conferences. This quality control structure continued throughout the 36-month intervention period.

***Intervention uptake and fidelity***

Given the pragmatic design where all eligible patients were automatically randomized to treatment arms, we closely tracked refusal rates and reasons for patients assigned to the Walk On! intervention. For participants who agreed to actively participate in Walk On!, we used the study dashboard to track uptake of the intervention components. Mild COPD exacerbations that are managed on an outpatient basis and hospitalizations/ED visits/observation stays for moderate to severe exacerbations are common in this cohort and were expected to be a major barrier to sustained PA and participation in intervention activities. Temporary suspension of intervention activities as requested by the patient or initiated by the coach due to COPD exacerbations or other acute illness as well as active withdrawals were documented. In order to balance the pragmatic nature of the study, we instituted a low intensity intervention fidelity assurance plan to include observations of up to five baseline intake visits and reviewing up to 5% of the planned and as needed telephone coaching contacts across each site.

## Measures and Outcomes

***Descriptive Variables***

*Socio-demographic variables:* Age, gender, marital status, education and income (census-based), race/ethnicity, and insurance status will be obtained from membership files

*Medications:* Pulmonary medications will be obtained from pharmacy databases. Supplemental oxygen use will be obtained from durable medical equipment files. Long-term oxygen use is defined as the patient being on oxygen >90 days, allowing a gap of no more than 14 days in the 12 months prior to cohort identification.

*Co-morbidities:* All available diagnoses from outpatient and inpatient encounters in the 12 months prior to cohort identification will be used to calculate the Charlson co-morbidity index

*Injurious Falls (DSMB Report):* The following E codes were used prior to 10/1/15: E880-E888. After 10/1/15, the following codes were used: W00-W19

***Primary Outcome***

The primary composite outcome is all-cause hospitalizations, emergency department (ED) visits, observational stays, and mortality in the 12 months following randomization. Given the multiple morbidities that patients with advanced COPD have and the known benefits of PA for these other chronic conditions, it is reasonable to expect that Walk On! will have positive effects on hospitalizations, ED visits, and observation stays for multiple causes. Walk On! is not expected to have its peak effects until at least 6 months into the program and thus, follow-up of at least 12 months is needed for all patients; and for those enrolled earlier, follow-up of up to 3 years will be available for secondary analyses of long term adherence and effectiveness.

**Table 3. Walk On! Data Collection Scheme**

| | **Pre-12 Months** | **Baseline** | **6 Months** | **12 Months** |
|---|---|---|---|---|
| **Primary Composite Outcome** | | | | |
| All-cause hospitalization | X | | | x |
| All-cause emergency department visits | X | | | x |
| All-cause observation stays | X | | | x |
| All-cause mortality | | | | x |
| **Secondary Outcomes** | | | | |
| COPD-related hospitalizations, ED visits, observation stays, exacerbations | X | | | x |
| Cardio-metabolic indicators (BMI, HbA1c, BP, and lipids) | X | | | x |
| *Patient-Reported Outcomes* | | | | |
| Self-reported physical activity (exercise vital sign) | x | x | x | x |
| COPD Assessment Test | | x | x | x |
| PROMIS-10 Quality of Life | | x | x | x |
| Personal Health Questionnaire, PHQ-9 | | x | x | x |
| Generalized Anxiety Disorder, GAD-7 | | x | x | x |
| Perception of Support for Exercise | | | x | x |
| Satisfaction with Walk On! | | | x | x |

***Secondary outcomes***

*COPD-related hospitalizations, ED visits, and observation stays* will be defined according to the current CMS criteria as detailed above in the description of the inclusion criteria.

*COPD exacerbations* will be ascertained via pharmacy records and utilization data. Mild to moderate exacerbations of COPD are typically characterized by changes in the current therapy to include increased use of bronchodilators, a short course of prednisone and/or antibiotics.[47,48] Our operational definition of an outpatient treated AECOPD included an in-person or virtual encounter (phone, email, or message) with or without a diagnosis of COPD (491.1, 491.21, 491.22, 491.9, 492, 492.8, 493.2, 493.22, and 496) documented with that encounter and accompanied by a prescription fill of either an oral steroid, ATB, or steroid and ATB within 2 days of the encounter.

| ICD-9 | ICD-10 |
|---|---|
| 491.1 | J41.1 |
| 491.21 | J44.1 |
| 491.22 | J44.0 |
| 491.9 | J42 |
| 492 | J43.1,J43.2,J43.8,J43.9 |
| 492.8 | J43.1,J43.2,J43.8,J43.9 |
| 493.2 | J44.0,J44.1,J44.9 |
| 493.22 | J44.1 |
| 496 | J44.9 |

A random sample of 185 probable AECOPD events were selected (n=15 records per strata) for chart review by two physicians; disagreements were adjudicated by HQN. Inter-rater reliability was assessed with a random 15% of the sample. Agreement between the two reviewers was excellent (kappa=0.93). Approximately 80% of the virtual encounters had a missing diagnosis code compared to 13% of the in-person clinic encounters. Restricting to only encounters that have a documented COPD diagnosis would fail to capture a large number of AECOPD events (sensitivity: 38%, specificity: 94%). The most optimal AECOPD definition which we propose to use included (1) encounters with a documented COPD diagnosis followed by a prescription fill of ATB, steroids, or ATB and steroids and (2) encounters with no documented COPD diagnosis but followed by a prescription fill of ATB and steroids (sensitivity: 67%, specificity: 84%)

*Physical Activity.* Every patient is asked two questions that capture their regular physical activity (exercise vital sign, EVS) during the intake process for all outpatient visits: 1) ‘‘On average, how many days per week do you engage in moderate to strenuous (vigorous) exercise (like a brisk walk)?’’ and 2) ‘‘On average, how many minutes do you engage in exercise at this level?’’ These questions are typically asked by front office staff, and patients’ responses are entered into the EMR. Response choices for days are categorical (0–7). Minutes are recorded as: 0, 10, 20, 30, 40, 50, 60, 90, 120, and 150 minutes or greater. The EMR system software then multiplies the two self-reported responses to display total minutes per week of moderate or vigorous physical activity (MVPA) for the health care provider to review. Due to the highly skewed MVPA data, we will categorize patients as being completely inactive (0 mins/week), insufficiently active (1-149 mins/week) or active, meeting national physical activity recommendations (≥150 mins/week). Patients with COPD in our health system have on average of 16 ambulatory visits over a year with approximately, 50% of those visits having usable EVS data. We will use all available EVS data to classify patients into their usual pattern of PA based on the modal/median EVS values. If a mode exists (most common category), then mode exercise category was used. If two exercise categories were equally the most common, then the higher category was recorded (unless categories are 0 and >150 min/wk then 1-150 min/wk category was used). The EVS has evidence of construct and predictive validity.[11,49,50]

*Cardio-metabolic Markers* include body mass index, systolic blood pressure, diastolic blood pressure, HbA1C, low density lipoprotein, high density lipoprotein, triglycerides, and total cholesterol. All

measurements available in the 12 months prior to identification will be averaged and used as baseline values.

For follow-up assessments of systolic and diastolic blood pressure, we will use the average of all routine clinic blood pressure readings taken between 6 and 12-months post-randomization. Blood pressures obtained with temperatures of $\geq$100F and those obtained in urgent care are excluded.

For the others, we will use the measure that is closest to the 12-month post-study enrollment date. Based on KPSC clinical care practices and our prior research experiences, we expect to have close to complete information on BMI and blood pressure for all patients; we should have near complete data on HbA1c for the approximately 35% of patients with co-morbid diabetes.

*Patient Reported Outcomes (PROs) (Appendix* Section 2*)*

*Health-Related Quality of life (HRQL).* Increased PA is expected to positively affect COPD and other co-morbid illnesses and consequently, improve patients' physical and mental health. The PROMIS-10 Global Quality of Life is used to measure HRQL.

*Symptoms.* COPD specific symptoms are measured with the COPD Assessment Test (CAT). Depression and anxiety which are common in COPD are assessed with the Personal Health Questionnaire, PHQ-9 and General Anxiety Disorder, GAD-7 survey. Note: The suicide ideation question of the PHQ-9 was removed after the second recruitment wave based on feedback from patients who felt the question was tangential to a program on physical activity. All study participants are referred to a depression care manager (if they agree) if they score 10 points or higher on the PHQ-8.

*Health behavior*. Physical activity, sedentary time, and sleep are measured using five questions modeled from national health surveys.

*Perception of support for PA* is measured with three questions which have been used our previous studies ask patients regarding the amount of support they receive for their physical activity from their coach, family members/friends, and health care provider.

*Satisfaction.* Overall satisfaction with Walk On! and its components including the baseline orientation, intensive follow-up in the first 4 weeks, pro-active monitoring, step goal setting using the IVR and Tractivity tools, reinforcement from the coach, and peer support will be measured at 6 and 12 months. We are also conducting semi-structured exit interviews with a randomly selected 25% of the Walk On! participants (or until thematic saturation) to understand the personal and ecological barriers and facilitators to successful uptake of Walk On!

**Table 4. Walk On! Data Summary**

| | **Source** |
|---|---|
| **Primary Composite Outcome** | |
| All cause death-hospitalizations-observation stays-emergency department visits | EMR, claims, membership files (death) |
| **Secondary Outcomes** | |
| COPD-related deaths-hospitalizations-observation stays-emergency department visits | EMR, claims, membership files (death) |
| Outpatient treated COPD exacerbations | EMR, pharmacy |
| Cardio-metabolic markers (body mass index, systolic blood pressure, | EMR |

| | |
|---|---|
| diastolic blood pressure, HbA1C, low density lipoprotein, high density lipoprotein, triglycerides, and total cholesterol) | |
| Health related quality of life (Physical and mental) | PROMIS-10 survey |
| Symptom burden | COPD Assessment Test |
| Depression | PHQ-8 |
| Anxiety | GAD-7 |
| Health behaviors | |
| Self-reported physical activity from routine care | EMR (exercise vital sign) |
| Physical activity history in 30's, 40's, 50's | Survey |
| Sedentary time (hrs) | Survey |
| Sleep (hrs) | Survey |
| Perception of support for physical activity | Survey |
| Satisfaction with Walk On! program components | Survey and semi-structured exit interviews |
| **Process Measures** | |
| Uptake/penetration of recruitment outreach | Study tracker |
| % patients agreed to participate | |
| % patients completed baseline visit (enrolled) | |
| Reasons for withdrawals, drop outs, & lost to F/U | Study tracker |
| % completed at least 4 coaching calls in first 5 weeks | Study dashboard |
| Total # phone contacts over 12 months (median, min, max) overall and by activity sensor used | Study dashboard |
| % participants attending group visits (1, 2-5, 6+) | Study tracker |
| Change in step counts over 12 months | Study dashboard |
| Physical activity coaches' perception of enablers and barriers to implementation of Walk On | Ongoing weekly coaches meeting |

## Analytical Plan

Descriptive statistics will be calculated prior to conducting the primary analyses. For all analyses, data consistency and assumptions required, e.g., normality of responses will be checked. Any data transformation or alternative methods necessary to analyze the data will be determined by examining the data structure. Baseline characteristics will be compared between the two groups to assess whether randomization balanced the group characteristics. The analyses will follow an intent-to-treat (ITT) strategy, i.e. the analyses will include all randomized participants in the groups to which they were randomly assigned, regardless of their adherence with the treatment and subsequent withdrawal.

### *Analysis for Aim #2*

To test the primary hypothesis that the proportion of patients with any occurrence of all-cause hospitalizations, ED visits, observation stays, and death 12 months after randomization will be significantly lower in the Walk On! intervention group compared to standard care, we will use logistic regression adjusted for randomization stratification variables (medical centers, time from hospitalization/ED/Ob Stay, level of activity and age). For the secondary outcomes, logistic regression will be used for categorical outcomes and analysis of variance will be used for continuous outcomes. Baseline characteristics that are unbalanced between the two groups will be included as covariates. Baseline characteristics for patients who do not complete the study due to health plan disenrollment will be compared to the patients who complete the study and differential "drop outs" between the two groups will be assessed by an interaction test between the intervention group and drop-out indicators. We expect little to no missing data for the health care utilization outcomes. Secondary as-treated analyses will be conducted based on actual treatment received to assess the efficacy of the intervention. Results from this analysis will be compared to the ITT analysis and any differences will be reported and interpreted with caution.

Since some participants will have follow-up data as far as 3 years after randomization, we will perform additional analyses to evaluate the intervention effect on long-term outcomes. We will use Poisson regression to assess the intervention effect on the average events during the entire study period and use survival analyses to assess the intervention effect on time to the first event. Generalized estimating equation (GEE) and mixed effects models will be used to compare the average proportions and mean changes for continuous outcomes between intervention groups, while taking into account correlated measures.

***Heterogeneity of treatment effect (Aim #3)***

We will assess heterogeneity of treatment effect by testing for a limited number of interactions, to determine whether intervention effects differ by patient subgroups, e.g. presence of other common morbidities (heart failure, diabetes, depression, and anxiety), level of social support, race/ethnicity (White vs. non-White), gender, age, and access to the Internet. These are pre-planned hypotheses and significant treatment heterogeneity will be declared through interaction tests with a standard alpha-level. The nature of the heterogeneity will be further assessed through subgroup analysis. Point estimates and appropriate confidence intervals will be presented. We may conduct other exploratory interaction and subgroup analyses, for which, appropriate alpha adjustment will be made to minimize the chance finding (type I error). Although these analyses will be exploratory in nature, it is critical that we understand what patient characteristics are associated with response to Walk On! in order to appropriately target the intervention in future dissemination efforts.

***Missing data***

Because of our integrated health delivery system and ability to capture all utilization internally and externally, we expect little to no missing data for the primary outcome or other secondary measures of health care utilization. For other EMR-based secondary measures such as self-reported physical activity and cardio-metabolic markers, we also expect to have nearly complete data since this patient cohort has on average 16 outpatient encounters with our health system annually. We will only analyze A1C and lipid data in the subset of patients with diabetes and cardiovascular disease. We expect a higher level of missing data for PROs (symptoms, quality of life). Baseline characteristics will be compared between patients with and without PRO data. For any missing data, we will assess whether data is likely missing completely at random (MCAR) or missing at random (MAR) or missing not at random (MNAR) by comparing patient characteristics. Sensitivity analysis and appropriate missing data imputation techniques will be deployed depending on the types of missing data. Results will be compared and differences will be interpreted with caution.

***Measuring and accounting for confounders***

All characteristics we can obtain from EMR and membership files will be extracted. This includes but is not limited to age, gender, marital status, insurance status, race/ethnicity, smoking status, BMI, Charlson index, O2 use, FEV1%predicted, prior number of hospitalization, etc. Due to randomization, we do not expect baseline values of these covariates will confound the data analysis assessing the effectiveness of Walk On! using the ITT samples. However, it is likely that these variables may confound the data analysis assessing the efficacy of Walk On! using the patients who actually receive the intervention (as-treated sample). This is because intervention acceptance, uptake and adherence may vary by patient characteristics and outcomes may vary by these characteristics. We will assess potential confounding and report results with and without appropriate adjustment.

***Qualitative analysis for Aim #4***

Interviews will be conducted with a random sample of active Walk On! participants at 6 month and 12 month (study completion) post-enrollment in both English and Spanish. These 30-minute telephone interviews will seek participant feedback about their interaction with coaches, most/least enjoyable aspects of participation, technical difficulties, improvements in health, and suggested study improvements.

Data will be collected by study staff ("interviewer") by telephone. The interviewer will type notes in to a Microsoft Word document during the interview. Immediately following the interview, the interviewer will review and improve upon the notes. Data will be compiled and pasted in to Microsoft Excel for analysis. Three project staff will independently code data based on eight thematic elements: most enjoyable aspect of program; least enjoyable aspect of program; will participant continue physical activity after the year of program participation; how long should the program last; technology; coach interaction; stamina/changes in health due to program; suggested program improvements. Staff will meet to review coding and resolve coding differences through discussion. Data will be triangulated with survey response data, support group session visits, and information gathered during coaches calls. Data will be reviewed by project staff and the Primary Investigator to determine saturation and identify areas for further clarification.

## Sample Size Estimates

***Primary composite outcome (hospitalizations/ED visit/observation stays/death)***

We have factored in features of this pragmatic study design such as randomizing all eligible patients and analyzing patients according to their group assignment regardless of the level of participation into our sample size calculations. Data from previous studies of self-management interventions in COPD showed a relative reduction of 30% in hospitalizations over 12 months of follow-up in volunteer sample with similar risk for hospitalizations.[51,52] Our previous observational findings showed that any level of moderate to vigorous PA was associated with a 34% reduction in 30-day all-cause readmissions for patients with an index COPD hospitalization.[11] Similarly, our more recent longitudinal analyses found that any level of PA was associated with a 38% reduction in mortality within 12-months after a COPD hospitalization.[50] In addition, our preliminary data suggest that nearly 50% of patients who had a COPD-related hospitalization, ED visit, or observation stay will have another hospital-based encounter, and a 20% will die in the subsequent 12 months.

Assuming that approximately 50% of the Walk On! patients participate in any aspect of the intervention, we estimated a conservative absolute reduction of 7% (relative reduction~10%) in the composite primary outcome of all-cause hospitalizations, ED visits, observation stays, and death. Allowing for a 15% disenrollment from the health plan and two-tailed α=.05, we anticipated that by enrolling a total of 1,650 patients, we will have 80% power to detect an absolute reduction of 7% in the primary composite outcome (70% vs. 63%).

*Note: Rationale for updated sample size target and power calculation approved by DSMB, PCORI and IRB in July 2016*

Assuming a revised target sample of n=2,700, allowing for 15% disenrollment and two-tailed α=.05, we will have 80% power to detect an effect as small as an absolute difference of 5.5% in the primary composite outcome of deaths, hospitalizations, observation stays, and ED visits between Walk On! and standard care (64.5% vs. 70%). Thus, with this revised target sample, we have adequate power to detect effect size that is smaller than our original proposed 7% absolute difference.

At this point, the target uptake rate that would translate to a minimum of 5.5% difference in the primary composite outcome between Walk On! and standard care in the intention to treat analysis is largely unknown. Based on the DSMB's review of the blinded Kaplan Meier curves for the study outcomes/adverse events thus far, the board thought it is possible that the average effects could be larger than our original proposed 7% absolute difference even with the uptake rate we have experienced so far in this trial. Given this uncertainty, the DSMB encouraged the team to continue to optimize our recruitment strategies and not to be particularly concerned about achieving an uptake threshold.

***Secondary EMR based outcomes***

For continuous EMR based outcomes, such as cardio-metabolic markers, this sample size will allow us to have at least 90% power to detect a small effect size of 0.20; the smallest effect size we can detect with 80% power is 0.16. We expect stronger intervention effects on the secondary outcomes of COPD-related events and proportion of inactive patients; thus, we have more than sufficient power to detect significant and clinically meaningful effects on these outcomes.

***Secondary patient-reported outcome (COPD Assessment Test, CAT)***

Our power calculation ($\alpha$=.05; $\beta$=.80) to detect a minimally clinically important difference in the primary PRO measure (COPD Assessment Test: $\Delta$ 2 points, SD: 5.2) showed that we need to have at least 112 completed 12-month survey responses. Factoring 20% attrition and a response rate of 45-60% using a combination of mail and telephone survey administration, we need to approach and administer the surveys to approximately 250 randomly selected standard care patients. Note: Change to sample.

## Safety Monitoring

The data and safety monitoring plan for this study included monitoring recruitment progress and potential adverse events resulting from data collection and the intervention activities. Since the pragmatic design precluded active outreach to the standard care patients, we relied on EMR-based data to conduct ongoing surveillance of events that resulted in a care encounter for safety monitoring. All serious adverse events related to study procedures were reported to the IRB and data safety monitoring board (DSMB).

## Project Milestones and Timeline

| **Table 5. Milestones and Timeline** | **Y1** | | | | **Y2** | | | | **Y3** | | | | **Y4** | | | |
|---|---|---|---|---|---|---|---|---|---|---|---|---|---|---|---|---|
| **Study Activities and Deliverables** | **Q 1** | **Q 2** | **Q 3** | **Q 4** | **Q 5** | **Q 6** | **Q 7** | **Q 8** | **Q 9** | **Q 10** | **Q 11** | **Q 12** | **Q 13** | **Q 14** | **Q 15** | **Q 16** |
| Project kick-off meeting | X | | | | | | | | | | | | | | | |
| IRB approval | X | | | | | | | | | | | | | | | |
| Pilot test Walk On! intervention at 4 medical centers | X | X | | | | | | | | | | | | | | |
| Develop & finalize intervention delivery tools in EMR | X | | | | | | | | | | | | | | | |
| Refine algorithms to identify participants from EMR | X | | | | | | | | | | | | | | | |
| Refine algorithms to ascertain outcomes from EMR | | X | | | | | | | | | | | | | | |
| Stakeholder meetings | | X | X | X | X | X | X | X | X | X | X | X | X | X | X | X |
| Data Safety Monitoring Board meetings | X | | | | X | | X | | X | | X | | X | | | |
| Identify and enroll new study participants | | | X | X | X | X | X | X | X | X | | | | | | |
| Implementation of Walk On! intervention | | | X | X | X | X | X | X | X | X | X | X | X | X | | |
| Process evaluation (formative and summative) | | | | X | X | X | X | X | X | X | X | X | X | X | | |
| Data collection and outcome ascertainment | | | | X | X | X | X | X | X | X | X | X | X | X | | |
| Data management, monitoring, QC, analysis | | | X | X | X | X | X | X | X | X | X | X | X | X | X | X |
| Dissemination: manuscripts and abstracts | | | | | | | | | | | X | X | X | X | X | X |

## REFERENCES


1. Minino A, Xu J, Kochanek K. *Deaths: Preliminary Data for 2008. National Vital Statistics Reports. National Center for Health Statistics.* Hyattsville, MD: Centers for Disease Control;2011.
2. Mannino DM, Homa DM, Akinbami LJ, Ford ES, Redd SC. Chronic obstructive pulmonary disease surveillance-United States, 1971-2000. *Morbidity and Mortality Weekly Report.* 2002;51(ss-6):1-16.
3. Booth S, Dudgeon D. *Dyspnea in Advanced Disease: A Guide to Clinical Management.* Oxford: Oxford University Press; 2006.
4. Booth S, Silvester S, Todd C. Breathlessness in cancer and chronic obstructive pulmonary disease: using a qualitative approach to describe the experience of patients and carers. *Palliat Support Care.* 2003;1(4):337-344.
5. Garcia-Aymerich J, Farrero E, Felez MA, Izquierdo J, Marrades RM, Anto JM. Risk factors of readmission to hospital for a COPD exacerbation: a prospective study. *Thorax.* 2003;58(2):100-105.
6. Pitta F, Troosters T, Probst VS, Spruit MA, Decramer M, Gosselink R. Physical activity and hospitalization for exacerbation of COPD. *Chest.* 2006;129(3):536-544.
7. Garcia-Aymerich J, Lange P, Benet M, Schnohr P, Anto JM. Regular physical activity reduces hospital admission and mortality in chronic obstructive pulmonary disease: a population based cohort study. *Thorax.* 2006;61(9):772-778.
8. Waschki B, Kirsten A, Holz O, et al. Physical activity is the strongest predictor of all-cause mortality in patients with chronic obstructive pulmonary disease: a prospective cohort study. *Chest.* 2011;140(2):331-342.
9. Moy ML, Teylan M, Weston NA, Gagnon DR, Garshick E. Daily step count predicts acute exacerbations in a US cohort with COPD. *PLoS One.* 2013;8(4):e60400.
10. Zanoria SJ, ZuWallack R. Directly measured physical activity as a predictor of hospitalizations in patients with chronic obstructive pulmonary disease. *Chron Respir Dis.* 2013;10:207.
11. Nguyen HQ, Chu L, Liu IL, et al. Associations between Physical Activity and 30-Day Readmission Risk in Chronic Obstructive Pulmonary Disease. *Ann Am Thorac Soc.* 2014;11(5):695-705.
12. Bradley EH, Sipsma H, Horwitz LI, Curry L, Krumholz HM. Contemporary Data About Hospital Strategies to Reduce Unplanned Readmissions: What Has Changed? *JAMA internal medicine.* 2013.
13. Brock J, Mitchell J, Irby K, et al. Association between quality improvement for care transitions in communities and rehospitalizations among Medicare beneficiaries. *JAMA.* 2013;309(4):381-391.
14. Hansen LO, Young RS, Hinami K, Leung A, Williams MV. Interventions to reduce 30-day rehospitalization: a systematic review. *Ann Intern Med.* 2011;155(8):520-528.
15. Spruit MA, Singh SJ, Garvey C, et al. An official american thoracic society/european respiratory society statement: key concepts and advances in pulmonary rehabilitation. *Am J Respir Crit Care Med.* 2013;188(8):e13-64.
16. Lacasse Y, Goldstein R, Lasserson TJ, Martin S. Pulmonary rehabilitation for chronic obstructive pulmonary disease. *Cochrane Database Syst Rev.* 2006(4):CD003793.
17. Johnston K, Grimmer-Somers K. Pulmonary rehabilitation: overwhelming evidence but lost in translation? *Physiother Can.* 2010;62(4):368-373.
18. Johnston KN, Young M, Grimmer KA, Antic R, Frith PA. Barriers to, and facilitators for, referral to pulmonary rehabilitation in COPD patients from the perspective of Australian general practitioners: a qualitative study. *Prim Care Respir J.* 2013.
19. Keating A, Lee A, Holland AE. What prevents people with chronic obstructive pulmonary disease from attending pulmonary rehabilitation? A systematic review. *Chron Respir Dis.* 2011;8(2):89-99.
20. Ries AL, Kaplan RM, Myers R, Prewitt LM. Maintenance after pulmonary rehabilitation in chronic lung disease: A randomized trial. *Am J Respir Crit Care Med.* 2003;167(6):880-888.

21. Pitta F, Troosters T, Probst VS, Langer D, Decramer M, Gosselink R. Are patients with COPD more active after pulmonary rehabilitation? *Chest.* 2008;134(2):273-280.
22. Mador MJ, Patel AN, Nadler J. Effects of Pulmonary Rehabilitation on Activity Levels in Patients With Chronic Obstructive Pulmonary Disease. *J Cardiopulm Rehabil Prev.* 2011;31(1):52-59.
23. Leidy NK, Kimel M, Ajagbe L, Kim K, Hamilton A, Becker K. Designing trials of behavioral interventions to increase physical activity in patients with COPD: insights from the chronic disease literature. *Respir Med.* 2014;108(3):472-481.
24. Vaes AW, Cheung A, Atakhorrami M, et al. Effect of 'activity monitor-based' counseling on physical activity and health-related outcomes in patients with chronic diseases: A systematic review and meta-analysis. *Ann Med.* 2013;45(5-6):397-412.
25. Berry MJ, Rejeski WJ, Miller ME, et al. A lifestyle activity intervention in patients with chronic obstructive pulmonary disease. *Respir Med.* 2010;104(6):829-839.
26. Steele BG, Belza B, Cain KC, et al. A randomized clinical trial of an activity and exercise adherence intervention in chronic pulmonary disease. *Arch Phys Med Rehabil.* 2008;89(3):404-412.
27. Liu WT, Wang CH, Lin HC, et al. Efficacy of a cell phone-based exercise programme for COPD. *Eur Respir J.* 2008;32(3):651-659.
28. Hospes G, Bossenbroek L, Ten Hacken NH, van Hengel P, de Greef MH. Enhancement of daily physical activity increases physical fitness of outclinic COPD patients: results of an exercise counseling program. *Patient Educ Couns.* 2009;75(2):274-278.
29. de Blok BM, de Greef MH, ten Hacken NH, Sprenger SR, Postema K, Wempe JB. The effects of a lifestyle physical activity counseling program with feedback of a pedometer during pulmonary rehabilitation in patients with COPD: a pilot study. *Patient Educ Couns.* 2006;61(1):48-55.
30. Nguyen H, Gill D, Wolpin S, Steele B, Benditt J. Pilot study of a cell phone-based exercise persistence intervention post-rehabilitation in COPD. *International Journal of COPD.* 2009;4:1-13.
31. Mendoza L, Horta P, Espinoza J, et al. Pedometers to enhance physical activity in COPD: a randomised controlled trial. *Eur Respir J.* 2015;45(2):347-354.
32. Nguyen HQ, Donesky D, Reinke LF, et al. Internet-based dyspnea self-management support for patients with chronic obstructive pulmonary disease. *J Pain Symptom Manage.* 2013;46(1):43-55.
33. Nguyen HQ, Donesky-Cuenco D, Wolpin S, et al. Randomized controlled trial of an internet-based versus face-to-face dyspnea self-management program for patients with chronic obstructive pulmonary disease: pilot study. *J Med Internet Res.* 2008;10(2):e9.
34. Moy ML, Weston NA, Wilson EJ, Hess ML, Richardson CR. A pilot study of an Internet walking program and pedometer in COPD. *Respir Med.* 2012;106(9):1342-1350.
35. Thorpe KE, Zwarenstein M, Oxman AD, et al. A pragmatic-explanatory continuum indicator summary (PRECIS): a tool to help trial designers. *CMAJ.* 2009;180(10):E47-57.
36. Adamson J, Cockayne S, Puffer S, Torgerson DJ. Review of randomised trials using the post-randomised consent (Zelen's) design. *Contemp Clin Trials.* 2006;27(4):305-319.
37. Zelen M. A new design for randomized clinical trials. *N Engl J Med.* 1979;300(22):1242-1245.
38. Bandura A. *Social foundations of thought and action. A social cognitive theory.* Englewood Cliffs, NJ: Prentice Hall; 1986.
39. Clark NM, Gong M, Kaciroti N. A model of self-regulation for control of chronic disease. *Health Educ Behav.* 2001;28(6):769-782.
40. Clark NM, Zimmerman BJ. A social cognitive view of self-regulated learning about health. *Health Education Research.* 1990;5(3):371-379.
41. Miller WR, Rollnick S. *Motivational Interviewing.* 2nd ed. New York: Guilford press; 2002.
42. Walker PP, Burnett A, Flavahan PW, Calverley PM. Lower limb activity and its determinants in COPD. *Thorax.* 2008;63(8):683-689.

43. Oka RK, King AC, Young DR. Sources of social support as predictors of exercise adherence in women and men ages 50 to 65 years. *Women's Health Research on Gender, Behavior, and Policy.* 1995;1(2):161-175.
44. Nguyen HQ, Steele BG, Dougherty CM, Burr RL. Physical Activity Patterns of Patients With Cardiopulmonary Illnesses. *Arch Phys Med Rehabil.* 2012.
45. Moy ML, Janney AW, Nguyen HQ, et al. Use of pedometer and Internet-mediated walking program in patients with chronic obstructive pulmonary disease. *J Rehabil Res Dev.* 2011;47(5):485-496.
46. Sallis R, Roddy-Sturm Y, Chijioke E, et al. Stepping toward discharge: Level of ambulation in hospitalized patients. *J Hosp Med.* 2015.
47. Anthonisen NR, Manfreda J, Warren CP, Hershfield ES, Harding GK, Nelson NA. Antibiotic therapy in exacerbations of chronic obstructive pulmonary disease. *Ann Intern Med.* 1987;106(2):196-204.
48. Rodriguez-Roisin R. Toward a consensus definition for COPD exacerbations. *Chest.* 2000;117(5 Suppl 2):398S-401S.
49. Liu IA, Moy ML, Estrada E, Rippberger E, Nguyen HQ. An 'Exercise Vital Sign' is a Valid Proxy Measure of Physical Activity in COPD in Routine Clinical Care. *Translational J of the American College of Sports Medicine.* 2017;2(23):148-152.
50. Moy ML, Gould MK, Liu IA, Lee JS, Nguyen HQ. Physical activity assessed in routine care predicts mortality after a COPD hospitalisation. *ERJ Open Res.* 2016;2(1).
51. Bourbeau J, Julien M, Maltais F, et al. Reduction of hospital utilization in patients with chronic obstructive pulmonary disease: a disease-specific self-management intervention. *Arch Intern Med.* 2003;163(5):585-591.
52. Rice KL, Dewan N, Bloomfield HE, et al. Disease management program for chronic obstructive pulmonary disease: a randomized controlled trial. *Am J Respir Crit Care Med.* 2010;182(7):890-896.
