# Supplementary material for: Effect of Physical Activity Coaching on Acute Care and Survival Among Patients With Chronic Obstructive Pulmonary Disease: A Pragmatic Randomized Clinical Trial
Source: JAMA Netw Open. 2019 Aug 16;2(8):e199657. doi: 10.1001/jamanetworkopen.2019.9657 (PMC6704745; doi:10.1001/jamanetworkopen.2019.9657)
Supplement: Supplement 2. — eMethods. Additional Statistical Analysis Information eTable 1. Additional Baseline Characteristics eTable 2. Walk On! Participants Process and Satisfaction Measures eTable 3. Time-to-Event Analyses of the Walk On! Intervention on the Primary Composite Outcome of All-Cause Hospitalizations, Observation Stays, Emergency Department Visits, and Death eTable 4. Cardio-metabolic Markers Post-randomization eFigure 1. Steps per Day Change Over 12 Months for Walk On! Participants Who Shared Their Data, by Level of Functioning at Baseline eFigure 2. Intent-to-Treat Adjusted Time-to-Event Analyses of the Walk On! Intervention on the Primary Composite Outcome of All-Cause Hospitalizations, Observation Stays, Emergency Department Visits, and Death 12-Months Post-randomization eFigure 3. As-Treated Adjusted Time-to-Event Analyses of the Walk On! Intervention on the Primary Composite Outcome of All-Cause Hospitalizations, Observation Stays, Emergency Department Visits, and Death from Months 2-12 Post-randomization [file jamanetwopen-2-e199657-s002.pdf]

Supplementary Online Content

Nguyen HQ, Moy ML, Liu I-LA, et al. Effect of physical activity coaching on acute care and survival among patients with chronic obstructive pulmonary disease: a pragmatic randomized clinical trial. *JAMA Netw Open.* 2019;2(8):e199657. doi:10.1001/jamanetworkopen.2019.9657

eMethods. Additional Statistical Analysis Information

eTable 1. Additional Baseline Characteristics

eTable 2. Walk On! Participants Process and Satisfaction Measures

eTable 3. Time-to-Event Analyses of the Walk On! Intervention on the Primary Composite Outcome of All-Cause Hospitalizations, Observation Stays, Emergency Department Visits, and Death

eTable 4. Cardio-metabolic Markers Post-randomization

eFigure 1. Steps per Day Change Over 12 Months for Walk On! Participants Who Shared Their Data, by Level of Functioning at Baseline

eFigure 2. Intent-to-Treat Adjusted Time-to-Event Analyses of the Walk On! Intervention on the Primary Composite Outcome of All-Cause Hospitalizations, Observation Stays, Emergency Department Visits, and Death 12-Months Post-randomization

eFigure 3. As-Treated Adjusted Time-to-Event Analyses of the Walk On! Intervention on the Primary Composite Outcome of All-Cause Hospitalizations, Observation Stays, Emergency Department Visits, and Death from Months 2-12 Post-randomization

This supplementary material has been provided by the authors to give readers additional information about their work.

© 2019 Nguyen HQ et al. *JAMA Network Open*.

eMethods. Additional Statistical Analysis Information

Power Calculations

Updated Power Calculation for Primary Outcome (approved by DSMB, PCORI and IRB in July 2016)

Assuming a revised target sample of n=2,700, allowing for 15% disenrollment and two-tailed α=.05, we will have 80% power to detect an effect as small as an absolute difference of 5.5% in the primary composite outcome of deaths, hospitalizations, observational stays, and ED visits between Walk On! and standard care (64.5% vs. 70%). Thus, with this revised target sample, we have adequate power to detect effect size that is smaller than our original proposed 7% absolute difference.

At the time of the revised power calculation, the target uptake rate that would translate to a minimum of 5.5% difference in the primary composite outcome between Walk On! and standard care in the intention to treat analysis was largely unknown. Based on the DSMB's review of the blinded Kaplan Meier curves for the study outcomes/adverse events at that time, the board thought it was possible that the average effects could be larger than our original proposed 7% absolute difference even with the uptake rate we experienced up to that point. Given this uncertainty, the DSMB encouraged the team to continue to optimize recruitment strategies and not to be particularly concerned about achieving an uptake threshold.

Power Calculation for the Primary PRO Measure (COPD Assessment Test, CAT): A total of 112 complete 12-month follow-up survey responses would be needed to detect a minimally clinically important difference between groups (CAT: Δ 2 points, SD: 5.2).

Statistical Analysis

Baseline characteristics in the year prior to the randomization were compared between groups using chi-square test for categorical variables, t-test or Wilcoxon rank-sum test for continuous variables where appropriate.

Analysis of variance or linear regression models were used to assess intervention effect on continuous outcomes, adjusting for baseline values.

Intent-to-treat multivariate analyses adjusted for randomization stratification variables (age, PA level, length of time since acute care utilization to randomization, and study site) as well as other prognostic variables (FEV1%predicted, Charlson comorbidity index, oxygen use, hospitalization for COPD in previous 12 months, outpatient treated COPD exacerbation in previous 12 months, and use of LABA or ICS).

We tested whether the intervention effects differed by pre-specified baseline characteristics (morbidities, level of social support, race/ethnicity, gender, age, and Internet access). These were examined by adding an interaction term in the models between the group indicator and the subgroup indicator and testing for significant interactions between the two.

© 2019 Nguyen HQ et al. *JAMA Network Open*.

eTable 1. Additional Baseline Characteristics[a]

Variables	Total Sample (n=2707)	Standard Care (n=1349)	Walk On! (n=1358)	P Value SC vs. WO	WO Participants (n=321)	WO Non-Participants (n=1037)	P Value WO P vs. Non- P
**Immunization**							
Influenza vaccination (prior year)	2369 (88%)	1180 (87%)	1189 (88%)	.95	286 (89%)	903 (87%)	.34
Pneumonia vaccination (up to 5 yrs)	1391 (51%)	681 (50%)	710 (52%)	.35	160 (50%)	550 (53%)	.32
**Pulmonary Rehabilitation** (up to past 3 yr)	169 (6%)	82 (6%)	87 (6%)	.72	27 (8%)	60 (6%)	.09
**Medications**							
Short-acting beta-agonist	2420 (92%)	1202 (92%)	1218 (92%)	.49	299 (94%)	919 (92%)	.23
Short-acting anticholinergic	1053 (40%)	525 (40%)	528 (40%)	.99	130 (41%)	398 (40%)	.74
Inhaled corticosteroids (ICS)	2147 (82%)	1094 (84%)	1053 (80%)	.02	264 (83%)	789 (79%)	.12
**Charlson Comorbidity Index**							
Quartile 1	1022 (38%)	506 (38%)	516 (38%)	.68	142 (44%)	374 (36%)	.06
Quartile 2	441 (16%)	210 (16%)	231 (17%)		53 (17%)	178 (17%)	
Quartile 3	680 (25%)	348 (26%)	332 (24%)		68 (21%)	264 (25%)	
Quartile 4	564 (21%)	285 (21%)	279 (21%)		58 (18%)	221 (21%)	
**Cardio-metabolic markers**[b]							
**Blood pressure**	n=2700	n=1347	n=1353		n=320	n=1033	
Systolic blood pressure	132.0 (12.7)	132.1 (12.9)	131.8 (12.5)	.60	131.2 (12.6)	132.0 (12.4)	.35
Diastolic blood pressure	71.2 (8.6)	71.4 (8.6)	71.1 (8.6)	.44	71.1 (8.2)	71.1 (8.7)	.86
**HbA1c (diabetics only)**	n=829	n=426	n=403		n=96	n=307	
	7.2 (1.2)	7.2 (1.2)	7.2 (1.2)	.21	7.2 (1.1)	7.3 (1.3)	.82
**Cholesterol**							
LDL (mg/dL)	n=1536	n=765	n=771	.80	n=194	n=577	.51
	91.7 (32.8)	92.0 (33.8)	91.3 (31.8)		90.1 (31.3)	91.8 (32.0)	
HDL (mg/dL)	n=1520	n=757	n=763	.51	n=189	n=574	.34
	54.9 (17.4)	55.1 (17.5)	54.6 (17.3)		55.2 (16.2)	54.4 (17.7)	
Total cholesterol (mg/dL)	n=1521	n=756	n=765	.55	n=190	n=575	.49
	169.1 (41.4)	170.3 (42.4)	168.0 (40.3)		166.6 (37.9)	168.5 (41.1)	
Triglycerides (mg/dL)	n=1031	n=506	n=525	.31	n=131	n=394	.08
	121.3 (77.4)	120.3 (77.1)	122.2 (77.8)		113.8 (86.2)	125.0 (74.7)	
**Body mass index (BMI)**	27.1 (5.9)	27.0 (6.0)	27.1 (5.9)	.66	27.5 (5.8)	27.0 (5.9)	.16

© 2019 Nguyen HQ et al. *JAMA Network Open*.

eTable 1. Additional Baseline Characteristics[a]

Variables	Total Sample (n=2707)	Standard Care (n=1349)	Walk On! (n=1358)	P Value SC vs. WO	WO Participants (n=321)	WO Non-Participants (n=1037)	P Value WO P vs. Non- P
Underweight (<18.5)	168 (6%)	93 (7%)	75 (6%)	.49	15 (5%)	60 (6%)	.53
Normal weight (18.5-24.9)	871 (32%)	426 (32%)	445 (33%)		97 (30%)	348 (34%)	
Overweight (25-29.9)	836 (31%)	419 (31%)	417 (31%)		105 (33%)	312 (30%)	
Obese (>30)	824 (31%)	408 (30%)	416 (31%)		103 (32%)	313 (30%)	
**Health Care Utilization in Prior Year**							
Primary care visits	6.1 (6.5)	5.9 (5.4)	6.2 (7.4)	.81	6.3 (4.8)	6.2 (8.1)	.01
Specialty care visits	9.1 (9.9)	9.3 (9.8)	8.9 (9.9)	.20	10.4 (11.6)	8.4 (9.2)	.002
Urgent care visits	1098 (41%)	560 (42%)	538 (40%)	.32	138 (43%)	400 (39%)	.16
	0.9 (1.7)	0.9 (1.6)	0.9 (1.8)	.42	0.9 (1.6)	0.9 (1.8)	.26
**All Cause**							
Hospitalizations	1.0 (1.4)	1.0 (1.4)	0.9 (1.3)	.13	0.9 (1.1)	1.0 (1.4)	.68
Observational stays	0.4 (0.8)	0.4 (0.8)	0.4 (0.8)	.79	0.4 (0.8)	0.4 (0.7)	.24
Emergency department visits	1.9 (3.1)	2.0 (3.2)	1.9 (3.0)	.24	1.5 (1.8)	2.0 (3.3)	.006
**COPD-Related**							
Hospitalizations	0.5 (0.8)	0.6 (0.8)	0.5 (0.7)	.07	0.5 (0.7)	0.5 (0.7)	.48
Observational stays	0.2 (0.5)	0.2 (0.5)	0.2 (0.4)	.64	0.2 (0.5)	0.2 (0.4)	.29
Emergency department visits	0.8 (1.0)	0.8 (1.0)	0.8 (1.0)	.38	0.7 (0.8)	0.8 (1.0)	.10
Outpatient treated COPD exacerbations	1.7 (2.0)	1.8 (2.0)	1.7 (1.9)	.05	1.9 (2.0)	1.6 (1.9)	.0007
**Patient Reported Outcomes**		n=268			n=320		
COPD Assessment Test (CAT)(↓0-40)	-	20.9(8.5)	-	.02	19.1(7.5)	-	-
CAT ≥10	-	242(90%)	-	.28	280(88%)	-	
PHQ-8 (↓0-24)	-	7.1 (6.0)	-	.05	5.9(5.2)	-	-
PHQ-8 ≥10	-	81 (31%)	-	<.01	65(21%)	-	-
GAD-7 (↓0-21)	-	5.7(5.9)	-	.32	4.8(4.9)	-	-
GAD-7 ≥10	-	61(23%)	-	.02	50(16%)	-	-
PROMIS-10 HRQL, Mental Health (21-68↑)	-	45.0(9.0)	-	.06	46.8(8.8)	-	-
PROMIS-10 HRQL, Physical Health (16-68↑)	-	39.2(8.6)	-	.03	40.1(6.8)	-	-
Sedentary time (hrs/day)[c]	-	5.5(4.1)	-	.04	4.8(3.0)	-	-

Note: Data are presented as either n(%) or mean(SD).

[a]Baseline values were obtained in the 12 months prior to cohort selection/randomization date

[b]Cardio-metabolic biomarkers include averages for all available BMI, BP, HbA1c, and lipids values over 12 months.

[c]Sedentary time: "In the last 7 days, please estimate the time you spent watching TV or videos on a typical day"

© 2019 Nguyen HQ et al. *JAMA Network Open*.

Abbreviations: HbA1c: Hemoglobin A1c; LDL: low density lipoprotein; HDL: high density lipoprotein; CAT: COPD assessment test; PROMIS-10 HRQL: Health-related quality of life; PHQ: Personal Health Questionnaire; GAD-7: Generalized Anxiety Disorder.

© 2019 Nguyen HQ et al. *JAMA Network Open*.

**eTable 2.** Walk On! Participants Process and Satisfaction Measures	**n (%) or median (IQR)**
**Walk On! Implementation**	
Participants completing at least 4 planned phone contacts, weeks 1-5	264 (83%)
Number of phone contacts/data reviews, weeks 6-52 for all patients[a]	12 (7, 20)
Omron pedometer users (n=177)	12 (6, 21)
Tractivity sensor users (n=81)	10 (6, 14)
Fitbit Alta users (n=13)	10 (3, 18)
Technical challenges: number of patients receiving at least 2 activity devices	72 (22%)
Participants attending at least one monthly group visit from 4 sites[b]	104 (42%)
**Overall satisfaction with Walk On![c]**	
Recommend Walk On! to other patients with COPD	
6 months (n=179)	175 (98%)
12 months (n=139)	138 (99%)
The Walk On! program was easy to fit into my life	
6 months (n=177)	165 (93%)
12 months (n=139)	135 (97%)

[a]Counts of documentation by the PA coaches on the study dashboard that either included an active phone contact or data review/no active contact. At the start of the study, patients could choose either the Tractivity internet-enabled sensor which is worn on the ankle or the Omron pedometer, worn on the waist. One year into the study, the Tractivity vendor was no longer in business. We were only able to provide the Omron pedometer to patients for six months while we configured our system to integrate the Fitbit Alta (worn on the wrist), as a replacement for the Tractivity sensor.
[b]Two sites had space challenges and did not offer group sessions; n=245 patients from 4 sites
[c]Endorsement of strongly agree or agree on a 4-point Likert scale (1-Strongly agree, 2-Agree, 3-Disagree, 4-Strongly disagree, 5-Not Applicable)

© 2019 Nguyen HQ et al. *JAMA Network Open*.

eTable 3. Time-to-Event Analyses of the Walk On! Intervention on the Primary Composite Outcome of All-Cause Hospitalizations, Observation Stays, Emergency Department Visits, and Death

	**Standard Care (n=1349)**	**Walk On! (n=1358)**	**Unadjusted HR**	**Adjusted HR**
**Primary intent-to-treat analysis: Follow-up for 12 months post randomization[a]**				
All-cause acute care utilization and death	864 (64%)	883 (65%)	1.00 (0.91,1.10)	1.01 (0.92, 1.11)
Hospitalizations	499 (37%)	502 (37%)	0.98 (0.87, 1.11)	1.01 (0.89, 1.15)
Observation stays	269 (20%)	295 (22%)	1.08 (0.91, 1.27)	1.08 (0.91, 1.27)
Emergency department visits	694 (51%)	702 (52%)	0.99 (0.89, 1.10)	1.01 (0.91, 1.12)
Death	117 (9%)	117 (9%)	0.97 (0.75, 1.26)	1.00 (0.78, 1.30)
COPD-related acute care utilization[d]	398 (30%)	411 (30%)	1.00 (0.87, 1.15)	1.04 (0.90, 1.19)
**Pre-specified, as-treated, IPTW analysis: Follow-up for months 2-12 post randomization[b]**				
	**Standard Care (n=1310)[c]**	**Walk On! Participants (n=321)**	**Unadjusted HR**	**Adjusted HR**
All-cause acute care utilization and death	781 (60%)	185 (58%)	0.94 (0.81, 1.10)	0.92 (0.79, 1.07)
Hospitalizations	433 (33%)	91 (28%)	0.80 (0.64, 0.99)	0.89 (0.73, 1.10)
Observation stays	230 (18%)	53 (17%)	0.92 (0.70, 1.22)	0.72 (0.53, 0.98)
Emergency department visits	610 (47%)	144 (45%)	0.93 (0.78, 1.10)	0.92 (0.77, 1.09)
Death	95 (7%)	13 (4%)	0.54 (0.30, 0.96)	0.70 (0.41, 1.19)
COPD-related acute care utilization[d]	195 (15%)	48 (15%)	0.94 (0.75, 1.18)	0.94 (0.75, 1.17)

HR indicates hazard ratio.
[a]Intent-to-treat: Adjusted OR values are from logistic regression models that included age, FEV1%predicted, Charlson comorbidity index, oxygen use, hospitalization for COPD in previous 12 months, outpatient treated COPD exacerbation in previous 12 months, length of time since acute care utilization to randomization, use of LABA or ICS, PA level, and study site.
[b]As-treated: stabilized propensity score inverse probability of treatment weighting (IPTW) was used to balance baseline characteristics (socio-demographics, health behaviors, disease severity, comorbidities, inhalers/medications, and health care utilization in the prior year) between patients who participated in Walk On! and the SC group.
[c]SC patients not included in the as-treated analysis due to disenrollment (n=17) and deaths (n=22) in the first 2 months after randomization.
[d]COPD-related acute care utilization includes hospitalizations, observational stays and ED visits for COPD exacerbations

© 2019 Nguyen HQ et al. *JAMA Network Open*.

**eTable 4.** Cardio-metabolic Markers Post-randomization					
		**Walk On**		**Adjusted P Values**	
	**SC**	**WO**	**WO Participants**	**SC vs. WO[c]**	**SC vs. WO-P[c]**
Blood pressure[a]	n=1127	n=1186	n=300		
Systolic blood pressure	130.8 (14.54)	130.7 (14.19)	128.8 (13.02)	0.70	0.06
Diastolic blood pressure	70.5 (9.59)	70.1 (9.63)	69.6 (8.85)	0.87	0.07
	n=412	n=383	n=97		
HbA1c (diabetics only)[b]	7.1 (1.45)	7.2 (1.36)	7.2 (1.40)	0.35	0.67
Cholesterol[b]					
	n=593	n=647	n=174		
LDL (mg/dL)	91.0 (36.96)	86.6 (32.51)	85.8 (29.92)	0.09	0.13
	n=620	n=671	n=178		
HDL (mg/dL)	54.3 (18.28)	53.1 (17.60)	52.8 (16.55)	0.82	0.34
	n=621	n=673	n=178		
Total cholesterol (mg/dL)	168.8 (44.91)	162.3 (41.82)	161.4 (38.31)	0.16	0.11
	n=372	n=429	n=114		
Triglycerides (mg/dL)	128.3 (102.36)	119.0 (65.60)	116.2 (61.36)	0.86	0.83

Data presented as mean (SD)

Abbreviations: HbA1c: Hemoglobin A1c; LDL: low density lipoprotein; HDL: high density lipoprotein;

[a]Blood pressure (BP): Average of all routine clinic BP readings taken between 6 and 12-months post-randomization. BP obtained with temperatures of >100F and those obtained in urgent care were excluded.

[b]HbA1c and cholesterol levels were obtained on values closest to the 12 months post randomization

[c]ITT and as-treated linear regression analyses adjusted for age, FEV1%predicted, Charlson comorbidity index, oxygen use, hospitalization for COPD in previous 12 months, outpatient treated COPD exacerbation in previous 12 months, length of time since acute care utilization to randomization, use of LABA or ICS, PA level, and study site. IPTW models provided similar results as the multivariate model.

© 2019 Nguyen HQ et al. *JAMA Network Open*.

eFigure 1. Steps per Day Change Over 12 Months for Walk On! Participants Who Shared Their Data, by Level of Functioning at Baseline

Mean step counts

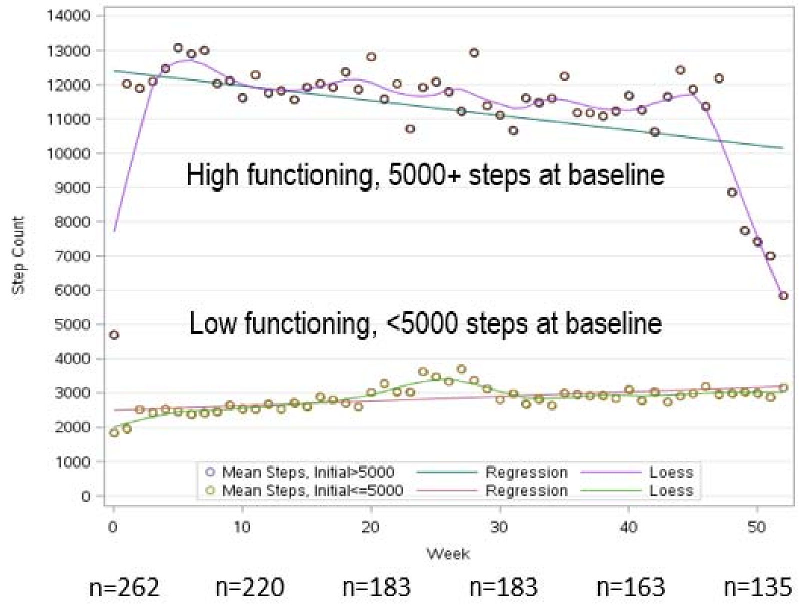

Median step counts

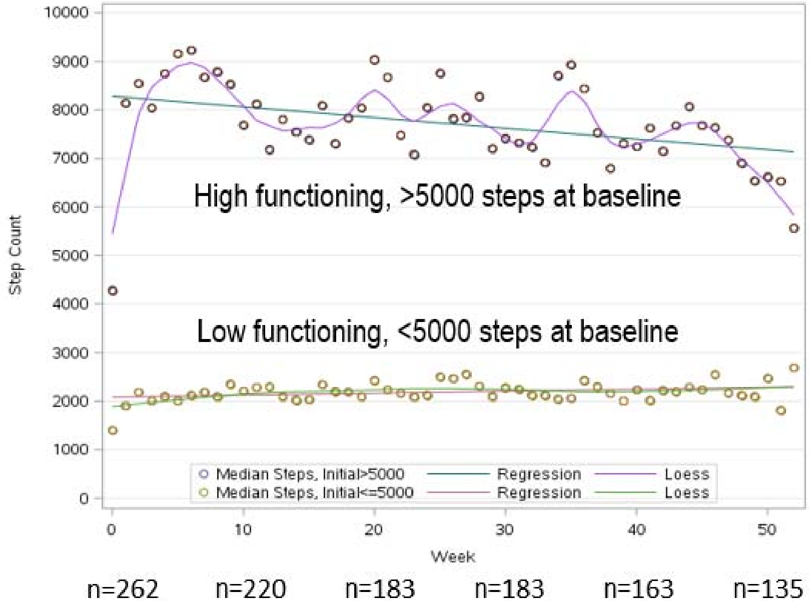

Note: Data in figure represents step count data that participants uploaded to their computer or entered into the phone interactive voice response system on a weekly basis. Only data uploads with non-zero values were included. Due to the heterogeneous sample and wide variation in baseline step count at baseline, mean and median step counts are presented, stratified by level of baseline "functioning", <5000 steps/day or 5000+ steps per day

© 2019 Nguyen HQ et al. *JAMA Network Open*.

eFigure 2. Intent-to-Treat Adjusted Time-to-Event Analyses of the Walk On! Intervention on the Primary Composite Outcome of All-Cause Hospitalizations, Observation Stays, Emergency Department Visits, and Death 12-Months Post-randomization

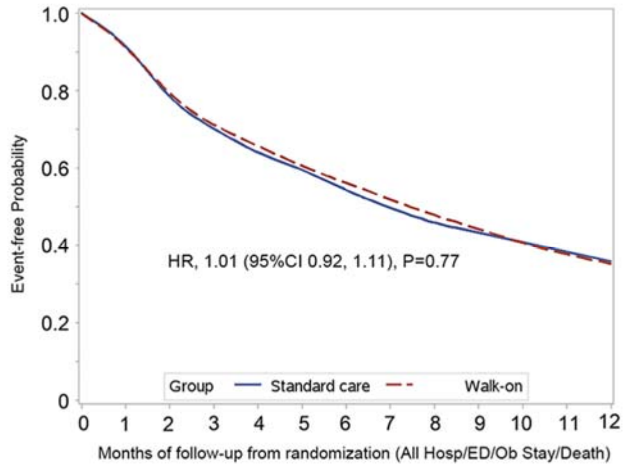

No at risk													
Standard Care	1349	1349	1228	1049	934	847	782	711	648	594	559	525	457
WO	1358	1358	1233	1069	956	877	807	747	689	631	583	533	461
Event													
Standard Care	0	115	289	400	481	541	609	669	719	753	785	818	864
WO	0	120	280	390	463	532	591	647	701	748	795	835	883

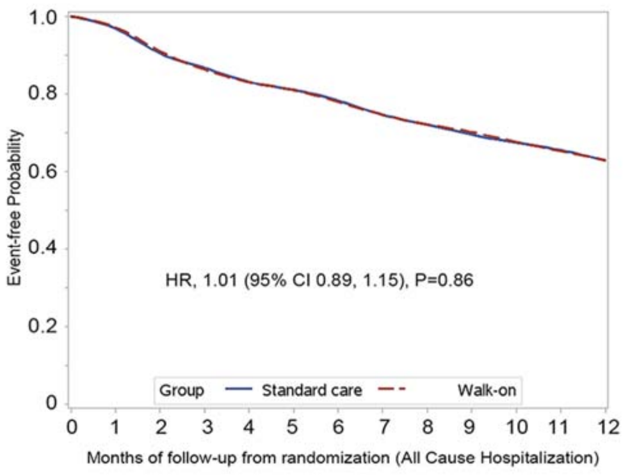

No at risk													
Standard Care	1349	1349	1296	1198	1138	1081	1044	1001	939	901	865	834	768
WO	1358	1358	1310	1217	1149	1099	1069	1025	975	935	905	866	794
Event													
Standard Care	0	42	127	177	224	250	286	332	364	396	422	446	499
WO	0	38	121	184	227	254	294	338	370	396	429	458	502

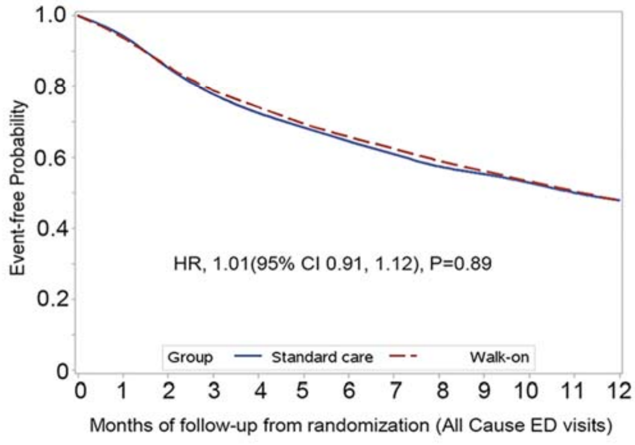

No at risk													
Standard	1349	1349	1263	1130	1023	944	883	828	773	722	691	657	586
WO	1358	1358	1263	1143	1045	975	912	863	814	765	724	680	603
Event													
Standard	0	77	197	295	365	417	468	515	558	586	615	650	694
WO	0	85	194	283	346	407	455	500	544	581	619	654	702

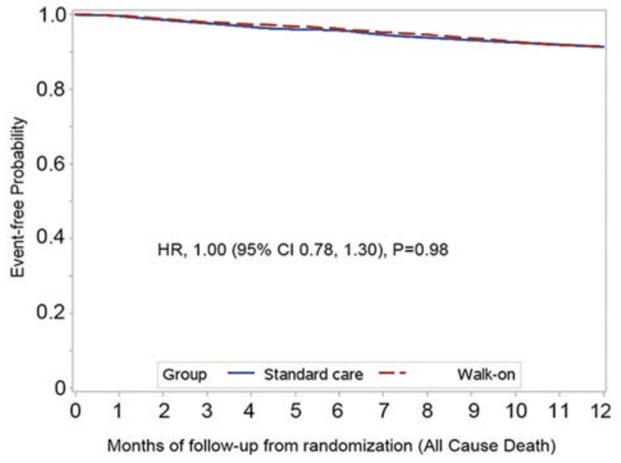

No at risk													
Standard Care	1349	1349	1335	1313	1295	1273	1254	1242	1215	1198	1183	1168	1134
WO	1358	1358	1348	1330	1316	1301	1291	1278	1258	1241	1227	1210	1181
Event													
Standard Care	0	6	19	31	45	53	57	71	81	90	98	105	117
WO	0	5	17	28	36	43	52	64	73	85	97	109	117

HR indicates hazard ratio. Number of events represents cumulative events and adjusted HRs and P values are from proportional hazards regression models that adjusted for age, FEV1%predicted, Charlson comorbidity index, oxygen use, hospitalization for COPD in previous 12 months, outpatient treated COPD exacerbation in previous 12 months, length of time since acute care utilization to randomization, use of LABA or ICS, PA level, and study site. Observation stays [HR, 1.08 (95% CI 0.91, 1.27), P=0.37] and COPD-related acute care utilization [HR, 1.04 (95% CI 0.90, 1.19), P=0.59] are not presented in the figures

© 2019 Nguyen HQ et al. *JAMA Network Open*.

eFigure 3. As-Treated Adjusted Time-to-Event Analyses of the Walk On! Intervention on the Primary Composite Outcome of All-Cause Hospitalizations, Observation Stays, Emergency Department Visits, and Death from Months 2-12 Post-randomization

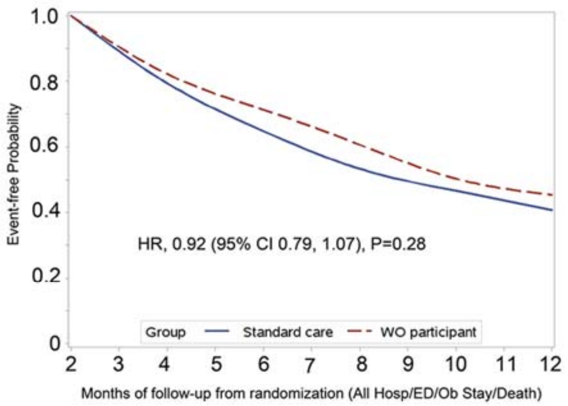

No at risk	2	3	4	5	6	7	8	9	10	11	12
Standard Care	1310	1310	1188	1029	924	831	743	676	628	586	507
WO Participants	321	321	296	264	241	229	206	194	174	160	144
Event											
Standard Care	0	116	268	367	456	539	602	647	687	726	781
WO Participants	0	25	57	80	92	114	126	145	159	166	185

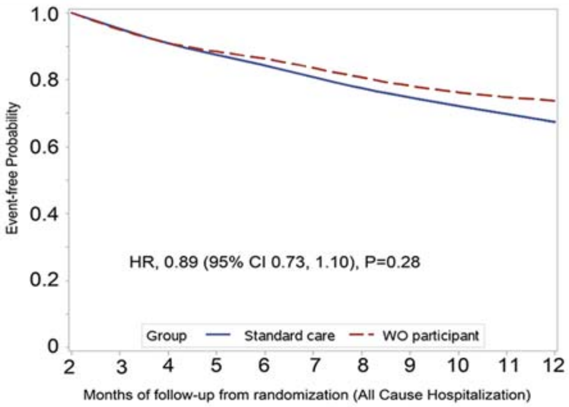

No at risk	2	3	4	5	6	7	8	9	10	11	12
Standard Care	1310	1310	1252	1165	1112	1061	993	952	914	878	809
WO Participants	321	321	308	291	282	276	264	257	248	242	231
Event											
Standard Care	0	44	118	159	202	252	287	321	351	377	433
WO Participants	0	13	29	38	44	55	62	70	76	82	91

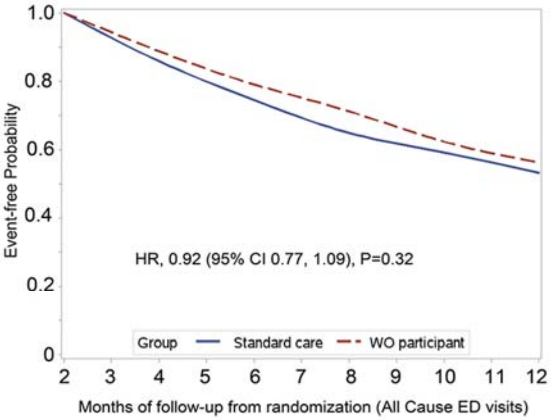

No at risk	2	3	4	5	6	7	8	9	10	11	12
Standard Care	1310	1310	1224	1100	1010	938	862	800	759	719	636
WO Participants	321	321	307	284	263	253	234	225	210	195	176
Event											
Standard Care	0	73	183	262	328	393	447	482	517	560	610
WO Participants	0	14	36	57	67	84	92	105	120	126	144

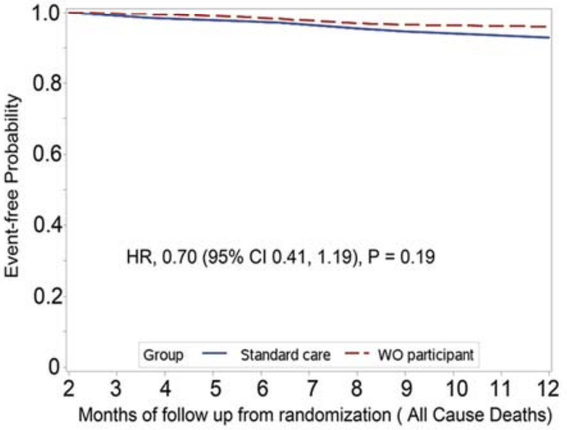

No at risk	2	3	4	5	6	7	8	9	10	11	12
Standard Care	1310	1310	1295	1273	1254	1242	1215	1198	1183	1168	1134
WO Participants	321	321	321	319	317	315	312	308	306	305	305
Event											
Standard Care	0	9	23	31	35	49	59	68	76	83	95
WO Participants	0	0	2	4	5	6	10	11	12	13	13

HR indicates hazard ratio. Number of events represents cumulative events and adjusted HRs and P values are from proportional hazards, stabilized propensity score inverse probability of treatment weighting (IPTW) regression models. Logistic regression was used to generate propensity scores using all available baseline characteristics (socio-demographics, health behaviors, disease severity, comorbidities, inhalers/medications, clinical biomarkers and health care utilization in the prior year) to balance the groups; SC patients not included in the as-treated analysis due to disenrollment (n=17) and deaths (n=22) in the first 2 months after randomization. Observation stays [HR, 0.72 (95% CI 0.53, 0.98), P=.04] and COPD-related acute care utilization [HR, 0.94 (95% CI 0.75, 1.17), P=.57] are not presented in the figures.

© 2019 Nguyen HQ et al. *JAMA Network Open*.
