# Supplementary material for: Effect of Physical Activity Coaching on Acute Care and Survival Among Patients With Chronic Obstructive Pulmonary Disease: A Pragmatic Randomized Clinical Trial
Source: JAMA Netw Open. 2019 Aug 16;2(8):e199657. doi: 10.1001/jamanetworkopen.2019.9657 (PMC6704745; doi:10.1001/jamanetworkopen.2019.9657)
Supplement: Supplement 3. — Data Sharing Statement [file jamanetwopen-2-e199657-s003.pdf]

Data Sharing Statement

Nguyen. Effect of Physical Activity Coaching on Acute Care and Survival Among Patients With Chronic Obstructive Pulmonary Disease. *JAMA Netw Open*. Published August 16, 2019. 10.1001/jamanetworkopen.2019.9657

Data
Data available: No

Additional Information
Explanation for why data not available: Data summaries will be shared upon request from principal investigator at the time of acceptance of publication of the study main findings. These summaries will be available to others without cost. The individual-level data set includes demographic characteristics, the various health conditions that each participant has, and a detailed account with dates of all utilization. Removal of all identifiers, to protect the identities of participants, will require significant time and costs.
